# Mechanisms of Antiproliferative Effects of Nobiletin, Scoparone, and Tangeretin Isolated from *Citrus reticulata* Peel Dichloromethane Extract in Acute Myeloid Leukemia Cells

**DOI:** 10.3390/ijms27031256

**Published:** 2026-01-27

**Authors:** Caterina Russo, Lutfun Nahar, Fyaz M. D. Ismail, Michele Navarra, Satyajit D. Sarker

**Affiliations:** 1 Department of Chemical, Biological, Pharmaceutical and Environmental Sciences, University of Messina, Viale F. Stagno d’Alcontres 31, 98166 Messina, Italy; carusso@unime.it; 2 Centre for Natural Products Discovery (CNPD), School of Pharmacy and Biomolecular Sciences, Liverpool John Moores University, James Parsons Building, Byrom Street, Liverpool L3 3AF, UK; profnahar@outlook.com (L.N.); f.m.ismail@ljmu.ac.uk (F.M.D.I.); s.sarker@ljmu.ac.uk (S.D.S.)

**Keywords:** *Citrus reticulata*, peel, nobiletin, scoparone, tangeretin, acute myeloid leukemia, cancer, apoptosis, mandarin, citrus waste

## Abstract

*Citrus reticulata* Blanco peel is a dominant industrial waste that was recently revalued as a source of bioactive molecules. This study explored its phytochemical and antileukemic potentials. The bioassay-guided fractionation of the dichloromethane extract yielded nobiletin, scoparone, and tangeretin, which were identified spectroscopically. The extract, fractions, and compounds showed antiproliferative effects in both THP-1 and U937 cells, which were employed as *in vitro* models of acute myeloid leukemia (AML). According to cytofluorimetric analysis, the extract and fractions inhibited cell growth by both apoptosis and necrosis, whereas the single molecules induced apoptosis. This mechanism was mediated by the modulation of *BAX* and *BCL-2* genes in both AML cell lines. Notably, each treatment drove THP-1 and U937 cells into the sub-G0 phase, together with an increase in the cell population in the G1 phase of the cell cycle, both of which were detected cytofluorimetrically. In line with these findings, the extract, fractions, and single molecules counteracted the overexpression of *CYCLIN A1* in THP-1 cells while reducing the expression of *CYCLIN D2* in U937 cells. Moreover, cell treatments attenuated the invasiveness of AML cells through the upregulation of *TIMP-2* at the transcriptional level. Therefore, this study supports pharmaceutical interest in citrus waste for cancer management, providing evidence on its antileukemic potential *in vitro*.

## 1. Introduction

*Citrus reticulata* Blanco (Rutaceae), from the Latin “reticulate” owing to the common net-like structure of its fruits, originated from South-East China and was then imported into Africa and Europe between the 15th and 19th centuries during Portuguese and British colonizations [1]. Nowadays, *C. reticulata* crops are widespread throughout the world, but are mainly cultivated in China, Brazil, Spain, Turkey, Morocco, Egypt, and Italy [2]. According to Linnaean classification, *C. reticulata* (mandarin) represents one of three ancestral species, along with *C. maxima* (pomelo) and *C. medica* (citron), of all existing citrus varieties [3]. Different varieties and hybrids, such as clementines, tangerines, satsumas, willow leaf, tangors, and tangelos, resulted from natural mutations of mandarins over time. Mandarins represent the second-most important citrus fruit cultivated globally, after oranges but ahead of lemons and grapefruits. They are appreciated for their sweet flavor, thick peel, and easy separation, accounting for 25% of global citrus production (about 37,000 tons per year) [2]. However, the high production of mandarins and their continued industrial processing generate a considerable amount of peel, which constitutes a dominant industrial waste and a serious issue for human and environmental health.

In the past, dried peels of *C. reticulata* were exploited in Traditional Chinese Medicine as a remedy for alleviating symptoms of indigestion, bronchitis, and asthma [4]. Recently, waste valorization practices, such as essential oil production or the recovery of added-value compounds, have been promoted to maximize the economic and health value of mandarin residues, such as peels [5]. In this regard, the isolation of appreciable amounts of polyphenolic compounds, including polymethoxyflavones (PMFs) and coumarins, has encouraged the evaluation of mandarin peel in the pharmaceutical field [6].

The dramatic life expectancies associated with some types of cancer have focused the efforts of the scientific community toward new strategies for cancer prevention and treatment. However, hematological malignancies still cause 6.3% of cancer-related deaths worldwide [7]. Among these, acute myeloid leukemia (AML) is considered one of the most aggressive forms in both children and adults because of its rapid progression and high genetic variability, with different molecular subtypes of the disease, such as AML-M5 (i.e., acute monocytic leukemia) [8]. The therapeutic limitations related to AML pathogenesis have prompted the search for alternative strategies, including the exploration of natural remedies for effective disease management.

*Citrus* fruits and their derivatives have gained widespread interest in the pharmaceutical field thanks to their well-recognized pharmacological properties, such as antioxidant, immunomodulatory, anti-inflammatory, neuroprotective, and anticancer effects [9,10,11]. In this regard, *C. reticulata* juice previously showed neuroprotective effects *in vitro* [12] and reduced the proliferation and migration of anaplastic thyroid carcinoma cells [13]. In relation to cancer, relevant evidence has been reported for *C. reticulata* peel.

Interestingly, *C. reticulata* peel exerted anticancer effects against aggressive forms of solid tumors, such as lung cancer [14], liver cancer [15], and pancreatic cancer [16]. In the field of hematological diseases, an aqueous *C. reticulata* peel extract and peel oil induced cytotoxicity in lymphoma cells and counteracted tumor development *in vivo* [6]. Moreover, mandarin peel exhibited measurable cytotoxic activity against leukemia HL-60 cells compared to grapefruit and lemon peels [17], although the role of *C. reticulata* peel in AML has not yet been fully elucidated.

From a waste valorization perspective, the aim of the present study was to define the phytochemical composition of *C. reticulata* peel and then to explore its antileukemic potential and that of one of its constituents in an *in vitro* model of AML, highlighting the underlying mechanisms of action.

## 2. Results

### 2.1. Crude Extracts of Citrus reticulata Peel

Soxhlet extraction of dried and ground peel of *C. reticulata* Blanco (mandarin) was performed in two batches (162.03 g and 155 g of powder), successively using *n*-hexane, dichloromethane (DCM), and methanol (MeOH) to obtain three extracts from each batch. The same solvent extracts from both batches were combined, for example, the two *n*-hexane extracts were combined to obtain one *n*-hexane extract (3.63 g). The combined weights and yields of the three resulting extracts are shown in Table 1.

**Figure 1:**
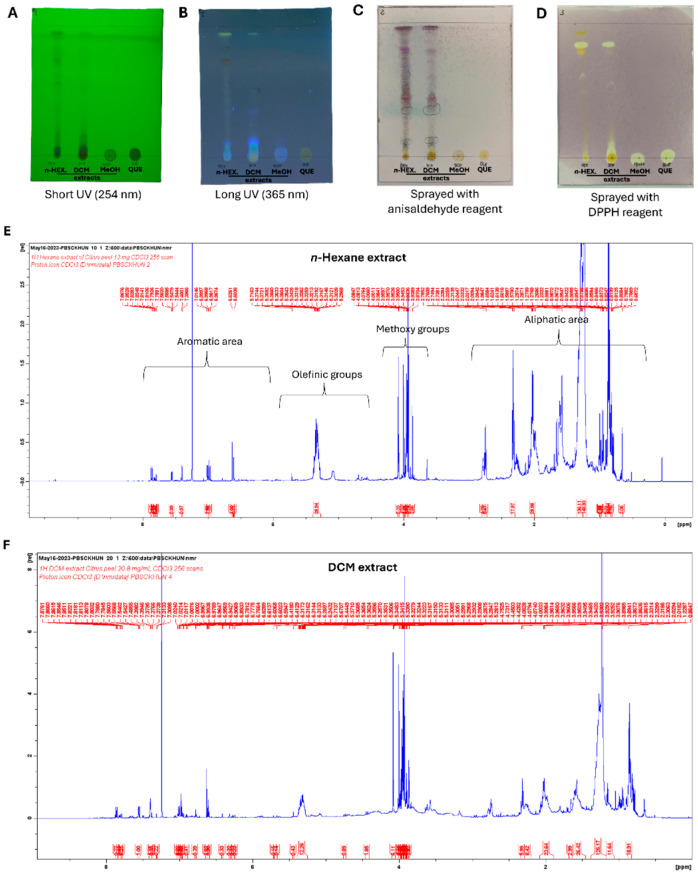
**Developed TLC plates of crude extracts from *C. reticulata* peel and ^1^H NMR spectra of its *n*-hexane and dichloromethane extracts.** *n*-Hexane, DCM, MeOH) extracts, and quercetin (QUE) were spotted on TLC plates and visualized under short-wavelength (**A**) and long-wavelength (**B**) UV light. The presence of violet, brown, and grey spots was observed in the developed TLC plate sprayed with anisaldehyde reagent (**C**). The presence of yellow spots was observed in another developed TLC plate after spraying with DPPH reagent (**D**). The ^1^H NMR spectra of *n*-hexane extract (**E**) and DCM extract (**F**) were recorded at 600 MHz using a Bruker AMX Ultrashield NMR spectrometer, with deuterium locking. Chemical shifts are reported in δ (ppm) with CDCl_3_ as the internal standard and coupling constants (*J*) are in Hz.

Then, the crude extracts were screened on a thin-layer chromatography (TLC) plate using a solvent system that allowed the separation of the *n*-hexane and DCM extracts, not the MeOH extract and quercetin (used as a positive control). In this regard, the *n*-hexane and DCM extracts displayed similar TLC patterns under UV light, suggesting the presence of alkanes, coumarins, and terpenes in the upper region, free fatty acids in the middle region, and phenolic compounds, including glycosylated flavonoids, in the lower region (Figure 1A,B). Similarly, the TLC plates of both extracts developed violet spots associated with the presence of triterpenes, brown spots due to diterpenes, and grey spots due to steroids when sprayed with anisaldehyde reagent (Figure 1C). Moreover, the TLC plates of both the *n*-hexane and DCM extracts developed yellow spots when sprayed with DPPH reagent, revealing antioxidant components with free-radical scavenging activity (Figure 1D).

The *n*-hexane and DCM extracts were analyzed via ^1^H NMR (600 MHz, CDCl_3_) to obtain a chemical fingerprint. The ^1^H NMR spectrum of the *n*-hexane extract (Figure 1A,E) showed signals attributable to aromatic ring protons (between δ_H_ 6 and 8 ppm), aliphatic hydrocarbons (between δ_H_ 0 and 3 ppm), methoxy groups (around δ_H_ 4 ppm), and olefinic groups (between δ_H_ 4.6 and 5.9 ppm). The ^1^H NMR spectrum of the DCM extract (Figure 1F) revealed the presence of a similar chemical fingerprint.

#### Antiproliferative Activity of *n*-Hexane and Dichloromethane Extracts in Acute Myeloid Leukemia Cells

**Figure 2:**
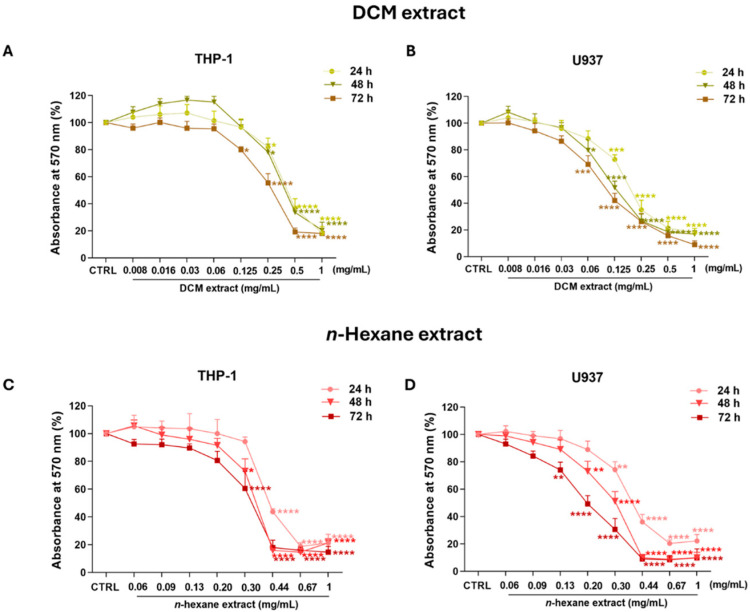
**Antiproliferative activity of dichloromethane and *n*-hexane extracts from *C. reticulata* peel in acute myeloid leukemia cells.** THP-1 (**on the left**) and U937 cell lines (**on the right**) were exposed to different concentrations of DCM (**A**,**B**) and *n*-hexane extracts (**C**,**D**) for 24, 48, and 72 h. Cell proliferation was assessed using the MTT assay. Results are expressed as absorbance percentage ± standard error of the mean (SEM). Values detected in treated cells are compared to those in control cells. Each experiment was carried out with eight replicates and repeated three times (*n* = 24). * *p* < 0.05, ** *p* < 0.01, *** *p* < 0.001, and **** *p* < 0.0001 vs. CTRL.

This study evaluated the potential antiproliferative activity of the *n*-hexane and DCM extracts in human monocytic THP-1 and human monoblastic U937 cells, i.e., *in vitro* models of AML-M5. In particular, THP-1 monocytes are a differentiated cell line of pediatric AML (i.e., AML-M5b), whereas U937 cells are immature monoblasts derived from adults (i.e., AML-M5a) [18]. As shown in Figure 2, treatment with the extracts already caused a significant reduction in cell growth after 24 h.

In detail, 0.25, 0.5, and 1 mg/mL concentrations of the DCM extract decreased THP-1 cell viability (about −20%, −65%, and −80%, respectively) after both 24 and 48 h, while a significant reduction in cell growth was also observed after 72 h of incubation with the 0.125 mg/mL concentration (−20.1 ± 2.2%, *p* < 0.05; Figure 2A). Interestingly, in U937 cells, the 0.125 mg/mL concentration of the DCM extract was effective after 24 h in U937 cells (−27.2 ± 3.5%, *p* < 0.001), suggesting the high sensitivity of this cell line compared to THP-1 cells. This was confirmed by evidence that, after 48 and 72 h of treatment, 0.06 mg/mL DCM extract induced a similar reduction in U937 cell viability as the 0.125 mg/mL concentration in THP-1 cells (Figure 2B).

Since no effect on cell growth was observed at concentrations of the *n*-hexane extract lower than 0.06 mg/mL, while small variations at higher concentrations caused large changes in cell proliferation, the interval of concentrations was set between 0.06 and 1 mg/mL. Indeed, treatment with 0.44 mg/mL *n*-hexane extract for 24 h drastically reduced THP-1 cell proliferation (−56.2 ± 2.1%; *p* < 0.0001), and a significant amount of cell growth was observed with 0.30 mg/mL after 48 and 72 h of incubation (Figure 2C). Even in this case, U937 cells showed a marked susceptibility to the treatment (Figure 2D).

For comparison, the IC_50_ values of both extracts are summarized in Table 2.

Since the chemical composition of the DCM extract was similar to that of the *n*-hexane extract, while its IC_50_ was lower, and considering its better solubility in culture medium/polar vehicle, further experiments were performed only using the former.

### 2.2. Fractionation of Dichloromethane Extract via Vacuum Liquid Chromatography

According to a bioassay-guided phytochemical analysis, the active DCM extract (1.44 g) was then fractionated. Fractionation was performed via vacuum liquid chromatography (VLC) on silica gel 60H, starting with elution using *n*-hexane–DCM mixtures and then increasing polarity with MeOH to obtain ten fractions (F1–F10). The weights of the ten fractions and related yields are presented in Table 3.

**Figure 3:**
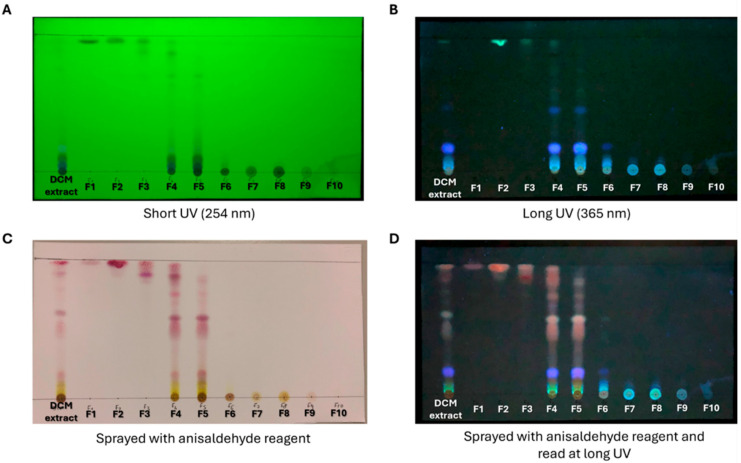
**Developed TLC plate of ten fractions obtained from dichloromethane extract.** DCM extract and its ten fractions (F1–F10) were spotted on a TLC plate and visualized under short-wavelength (**A**) and long-wavelength (**B**) UV light. The presence of brown, yellow, green, and violet spots was detected after spraying the TLC plate with anisaldehyde reagent (**C**) and then observed under UV light (**D**).

The chemical composition of each DCM fraction was screened on a TLC plate under UV light (at 254 nm, Figure 3A; at 365 nm Figure 3B). The TLC plate sprayed with anisaldehyde reagent (Figure 3C) revealed under UV light the presence of diterpenes/tannins (brown spots), flavonoids (yellow spots), sesquiterpenes (phosphorous green-blue spots), and sugars/triterpenes (violet spots) within different fractions (Figure 3D). In particular, the F4 and F5 DCM fractions seemed to be the richest in the abovementioned compounds, as well as the most representative of the DCM extract (68% of the total extract weight; Figure 3 and Table 3). Therefore, the F4 and F5 fractions, which possessed similar TLC fingerprints, were combined (F_DCM_).

#### Antiproliferative Activity of Combined Dichloromethane Fractions in Acute Myeloid Leukemia Cells

**Figure 4:**
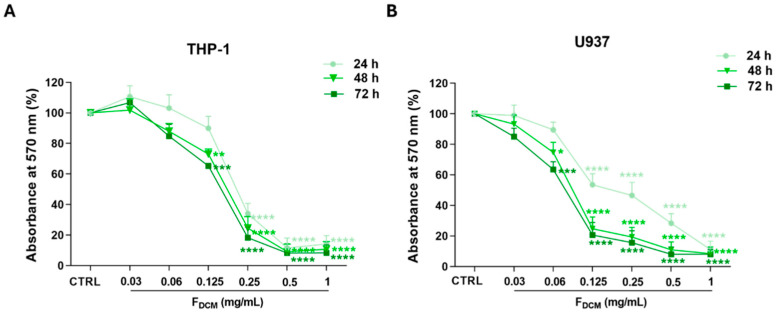
**Antiproliferative activity of combined dichloromethane fractions (F_DCM_) in acute myeloid leukemia cell lines.** THP-1 (**A**) and U937 (**B**) cell lines were exposed to increasing concentrations of F_DCM_ for 24, 48, and 72 h. Cell proliferation was evaluated using the MTT assay. Results are expressed as absorbance percentage ± standard error of the mean (SEM). Values detected in treated cells are compared to those in control cells. Each experiment was carried out with eight replicates and repeated three times (*n* = 24). * *p* < 0.05, ** *p* < 0.01, *** *p* < 0.001, and **** *p* < 0.0001 vs. CTRL.

Fractions 4 (F4) and 5 (F5) of the DCM extract were combined (F_DCM_) and the antiproliferative activity of F_DCM_ was tested in leukemia cell lines. Interestingly, treatment with F_DCM_ was more effective than treatment with the DCM extract in THP-1 cells. As shown in Figure 4A, the 0.125 mg/mL concentration significantly reduced the growth of THP-1 cells after 48 h (−26.9 ± 3.3%, *p* < 0.01) and 72 h (−34.7± 1.6%). As expected, F_DCM_ also decreased the cell proliferation of U937 cells to a greater extent than the DCM extract (Figure 4B).

A comparison between the antiproliferative activity of F_DCM_ and the DCM extract is displayed in Table 4.

### 2.3. Isolation of Compounds via Preparative Thin-Layer Chromatography

The main compounds of F_DCM_ were isolated via preparative thin-layer chromatography (PTLC) and identified using spectroscopic techniques.

**Figure 5:**
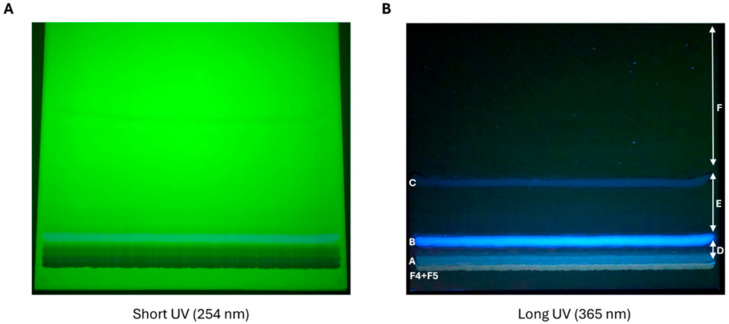
**Chromatographic separation of combined F4 and F5 dichloromethane fractions (F_DCM_) via preparative TLC.** Thin bands of F_DCM_ were applied to a silica gel plate. The developed TLC plate was visualized under short-wavelength (**A**) and long-wavelength (**B**) UV light. Three bands (A, B, and C bands in light blue) were separated, and other potential mixtures of compounds were retained in D, E, and F spaces between bands.

PTLC was performed to separate the mixture of the two DCM fractions (F_DCM_). Briefly, 20.5 mg was dissolved in DCM to prepare a solution of 10 mg/mL, and at least five silica gel plates were used to obtain a satisfactory separation. The developed TLC plates were visualized under UV light (Figure 5A). Then, separated bands (namely A, B, and C; Figure 5B) as well as the spaces between bands (namely D, E, and F; Figure 5B) were individually collected for further processing. The weights and yields of the separated samples are reported in Table 5.

**Figure 6:**
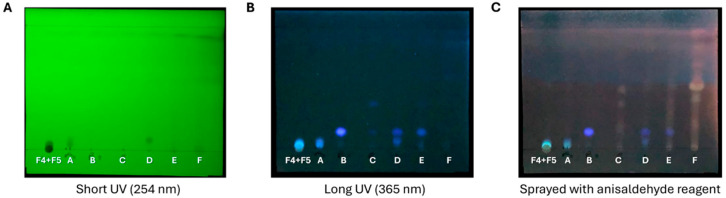
**Developed TLC plate of A–F samples separated from combined dichloromethane fractions (F_DCM_).** Samples A–F were spotted on a TLC plate, which was visualized under UV light at a short wavelength (**A**) and at a long wavelength (**B**). The purity of samples was further evaluated on a TLC plate after spraying with anisaldehyde reagent and visualization at 365 nm (**C**).

The purity of isolated samples A–F was then investigated via analytical TLC (Figure 6A). Only samples A and B appeared to be pure on the TLC plate. However, sample D appeared to be partially impure on the TLC plate, being partly contaminated by traces of compound A, as revealed by their similar spots (Figure 6B) and close separation on the silica gel plate. Other isolated samples (i.e., C, E, and F) were found to be impure on the TLC plate after spraying with anisaldehyde reagent (Figure 6C).

Based on preliminary TLC screening of samples A–F, this study focused on almost pure compounds, such as A, B, and D, for identification using spectroscopic techniques. For non-pure samples C, E, and F, no further spectroscopic analyses were performed.

### 2.4. Identification of Compounds A, B, and D via Spectroscopic Analysis

**Figure 7:**
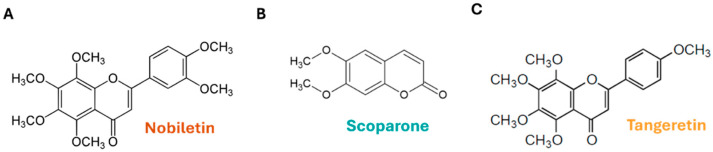
**Compounds identified in combined dichloromethane fractions (F_DCM_).** The chemical structures of the polymethoxyflavone nobiletin (**A**); 6,7-dimethoxycoumarin, namely scoparone (**B**); and 3′-demethoxy nobiletin, known as tangeretin (**C**) are shown.

1D NMR analysis was performed on samples A, B, and D isolated from F_DCM_, leading to the identification of three known compounds, namely nobiletin (NOB, Figure 7A; ^1^H NMR spectrum of NOB is reported in Figure S1), scoparone (SCO, Figure 7B; ^1^H NMR spectrum of SCO is reported in Figure S2), and tangeretin (TAN, Figure 7C; ^1^H NMR spectrum of TAN is reported in Figure S3). The spectral data of these compounds were comparable to published data for the respective compounds (for NOB and TAN [19]; for SCO [20]).

#### Antiproliferative Activity of Isolated Compounds in Acute Myeloid Leukemia Cells

**Figure 8:**
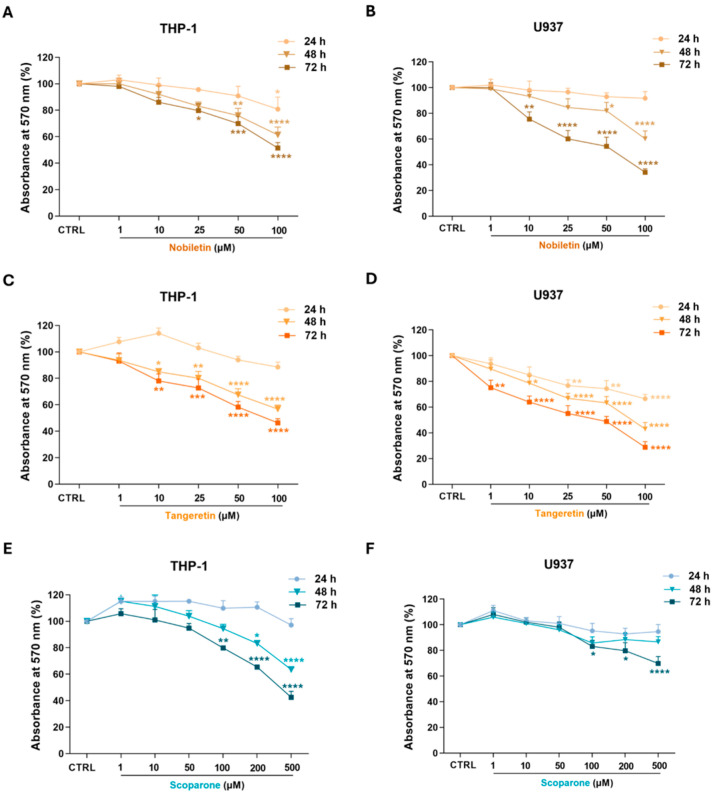
**Antiproliferative activity of nobiletin, tangeretin, and scoparone in THP-1 and U937 leukemia cells.** THP-1 (**on the left**) and U937 (**on the right**) cell lines were exposed to increasing concentrations of nobiletin (**A**,**B**), tangeretin (**C**,**D**), and scoparone (**E**,**F**) for 24, 48, and 72 h. Cell proliferation was evaluated using the MTT assay. Results are expressed as absorbance percentage ± standard error of the mean (SEM). Values detected in treated cells are compared to those in untreated cells. Each experiment was carried out in eight replicates and repeated three times (*n* = 24). * *p* < 0.05, ** *p* < 0.01, *** *p* < 0.001, and **** *p* < 0.0001 vs. CTRL.

Thus, the isolated compounds identified as NOB, TAN, and SCO were assessed for their antiproliferative activity in AML cells. In this regard, the antiproliferative effects of 50 and 100 µM NOB in THP-1 cells were significant after 48 h of incubation, and in the range of 25–100 µM after 72 h of treatment (Figure 8A). Also, in the case of the single compounds, U937 cells were more sensitive than THP-1 cells to NOB, which significantly reduced cell growth after incubation with 10 µM for 72 h (−24.4 ± 5.6%, *p* < 0.01; Figure 8B).

Treatment with TAN (Figure 8C) significantly reduced the cell proliferation of THP-1 cells within the range 10–100 µM (from −15.1 ± 2.5%, *p* < 0.05, to −43.2 ± 3%, *p* < 0.0001, at 48 h; from −21.9± 5.3%, *p* < 0.01, to −53.7± 3.1%, *p* < 0.0001, at 72 h of incubation). Consistently, the growth of U937 cells was significantly inhibited by TAN (25–100 µM), as early as 24 h. This effect was amplified at 48 and 72 h, leading to a decrease in cell growth by 56.9 ± 5% and 71.1 ± 4.4% (*p* < 0.0001) with 100 µM. After 72 h of treatment, TAN concentrations of 1 µM and 10 µM were also effective in reducing cell proliferation by 24.9 ± 5.8% (*p* < 0.01) and 36 ± 4.7% (*p* < 0.0001), respectively (Figure 8D).

SCO demonstrated less activity because its antiproliferative activity in THP-1 cells started at the 100 µM concentration after 72 h of incubation (−20.1± 1.4%, *p* < 0.01; Figure 8E). Unlike NOB and TAN, the antiproliferative activity of SCO in U937 cells was less than that in THP-1 cells, as SCO was only able to reduce cell growth by 30.1 ± 5.3% (*p* < 0.0001) with the 500 µM concentration after 72 h of incubation (Figure 8F).

Table 6 presents the IC_50_ values of the antiproliferative activity of the single compounds, which guided the choice of concentrations to employ in further *in vitro* investigations aimed at elucidating the mechanisms underlying the reduction in cell growth.

### 2.5. Apoptosis Assessment

To discriminate between apoptotic and necrotic cell death, this study performed the Annexin V/propidium iodide staining assay. Then, the study investigated the molecular mechanisms underlying cell death via RT-PCR.

#### 2.5.1. Dichloromethane Extract and Fractions Induced Apoptosis and Necrosis in Acute Myeloid Leukemia Cells

**Figure 9:**
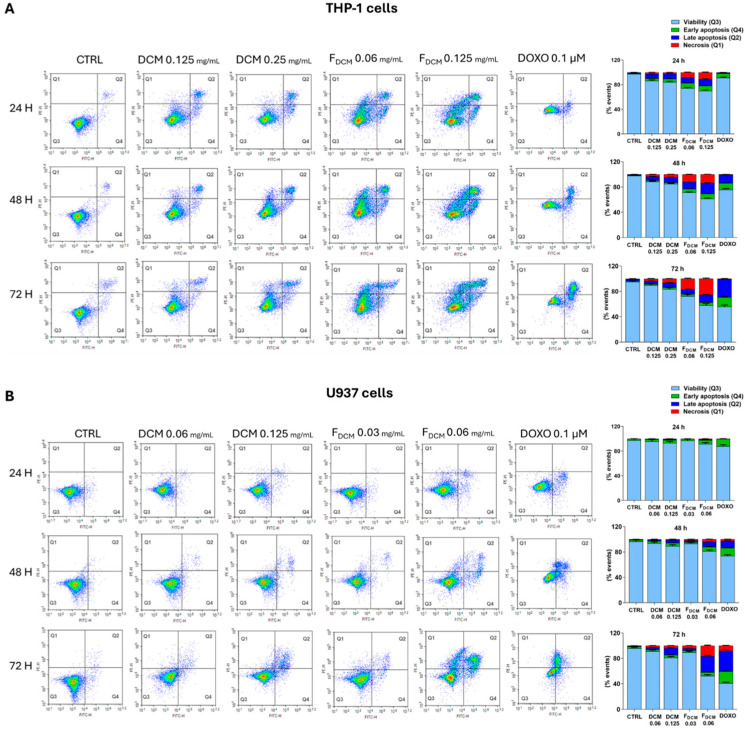
**Cell death mechanisms induced by dichloromethane (DCM) extract and fraction (F_DCM_) in acute myeloid leukemia cells.** Leukemia THP-1 (**A**) and U937 cells (**B**) were exposed to increasing concentrations of DCM extract or F_DCM_ for 24, 48, and 72 h, or to doxorubicin. Assessment of apoptosis was performed using the Annexin V-FITC/propidium iodide staining assay. On the left, representative density plots (Annexin V vs. PI) are shown. Each plot is subdivided into four quadrants: containing viable cells in Q3, cells in early apoptosis in Q4, cells in late apoptosis in Q2, and cells in necrosis in Q1. Histograms shown on the right express the percentage of cells for each quadrant (Q3: light blue bars; Q4: green bars; Q2: blue bars; Q1: red bars) ± SEM of three experiments performed in triplicate (*n* = 9).

As shown in Figure 9A, the treatment of THP-1 cells with 0.25 mg/mL DCM extract caused a weak increase in the apoptotic population (10 ± 0.7% after 72 h) as well as in the necrotic population (6.3 ± 0.4% after 72 h). F_DCM_ was more effective than the DCM extract since it induced apoptosis at the 0.06 mg/mL concentration after 24 h (17.1 ± 0.8%) and, in parallel, it increased the population of necrotic cells in a time- and concentration-dependent fashion.

Based on the IC_50_ values, this study tested lower concentrations of the DCM extract and F_DCM_ in U937 cells than in THP-1 cells (Figure 9B). In this regard, a slight increase in the population of apoptotic cells was observed when the culture was treated with 0.125 mg/mL DCM extract for 48 h (9.5 ± 0.6%), which rose to 15 ± 0.7% after 72 h of incubation, while necrotic cells comprised 3.4 ± 0.3% of cells. The same results observed when cells were exposed to 0.125 mg/mL DCM extract for 72 h were replicated after 48 h of treatment with 0.06 mg/mL F_DCM_, while the study detected 31.1 ± 1.7% apoptotic cells and 16.4 ± 0.9% necrotic cells after 72 h. Finally, doxorubicin, which was used as positive control, caused apoptotic cell death in both AML cell lines, with slight but not significant necrosis.

#### 2.5.2. Induction of Apoptosis by Nobiletin, Tangeretin, and Scoparone in Acute Myeloid Leukemia Cells

**Figure 10:**
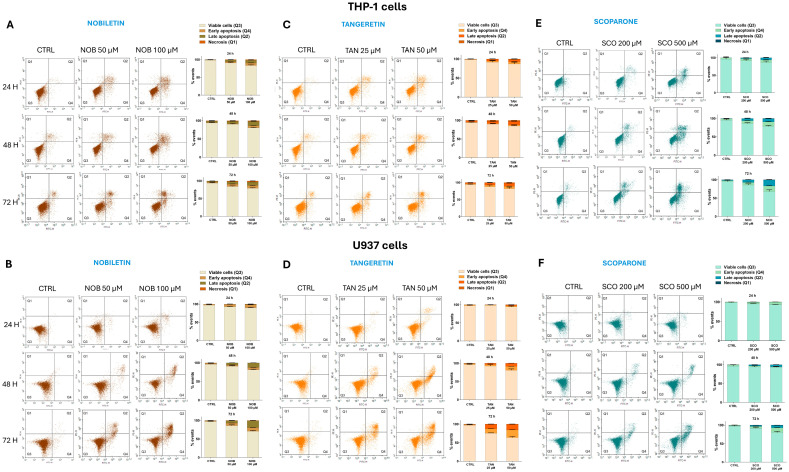
**Cytofluorimetric evaluation of apoptosis in acute myeloid leukemia cells exposed to nobiletin, tangeretin, and scoparone.** The assessment of apoptosis was performed using the Annexin V/propidium iodide assay. Representative Annexin V vs. PI dot plots, along with related quantitative graphs, are shown. THP-1 cells (upper) and U937 cells (lower) were treated with different concentrations of nobiletin (NOB, (**A**,**B**)), tangeretin (TAN, (**C**,**D**)), or scoparone (SCO, (**E**,**F**)) for 24, 48, and 72 h. Each plot is divided into four quadrants, containing viable cells in Q4, cells in early apoptosis in Q3, cells in late apoptosis in Q2, and cells in necrosis in Q1. Histograms next to plots report the percentages of cells for each quadrant *±* SEM of three independent experiments performed in triplicate (*n* = 9). The negative control (CTRL) for THP-1 cells treated with NOB and TAN is the same, so CTRL plots shown in (**A**,**C**) are the same. Similarly, experiments on U937 cells were performed using the same negative control, so CTRL plots in (**B**,**D**,**F**) are identical.

The study investigated the cell death caused by NOB, TAN, and SCO in leukemia cells. Figure 10A shows that the number of THP-1 cells in apoptosis increased when treated with 50 µM NOB and increased even more when treated with 100 µM NOB, accounting for 13.4 ± 0.7% and 17.3 ± 0.9% of cells after 72 h of treatment, respectively. However, the pro-apoptotic effect of NOB in THP-1 cells appeared to be less intense than that in U937 cells. In this regard, the numbers of cells undergoing apoptosis were 13.3 ± 0.9% and 28.2 ± 1.5% after 72 h of treatment with 50 and 100 µM NOB, respectively (Figure 10B).

Exposure of THP-1 cells to 50 µM TAN for 72 h induced apoptosis in 15.5 ± 0.8% of cells (Figure 10C), which increased further in U937 cells, reaching 35.6 ± 1% (Figure 10D).

In line with the data from the proliferation assay, SCO was more effective against THP-1 cells than against U937 cells (Figure 10E). In detail, 500 µM SCO caused apoptosis in 31.3 ± 3.2% of THP-1 cells after 72 h of exposure, whereas apoptosis in U937 cells reached 19 ± 1.4% (Figure 10F). Finally, NOB, TAN, and SCO did not induce necrotic cell death.

#### 2.5.3. Dichloromethane Extract, Fractions, and Single Compounds Modulated the Expression of Apoptotic-Related Genes in Acute Myeloid Leukemia Cells

**Figure 11:**
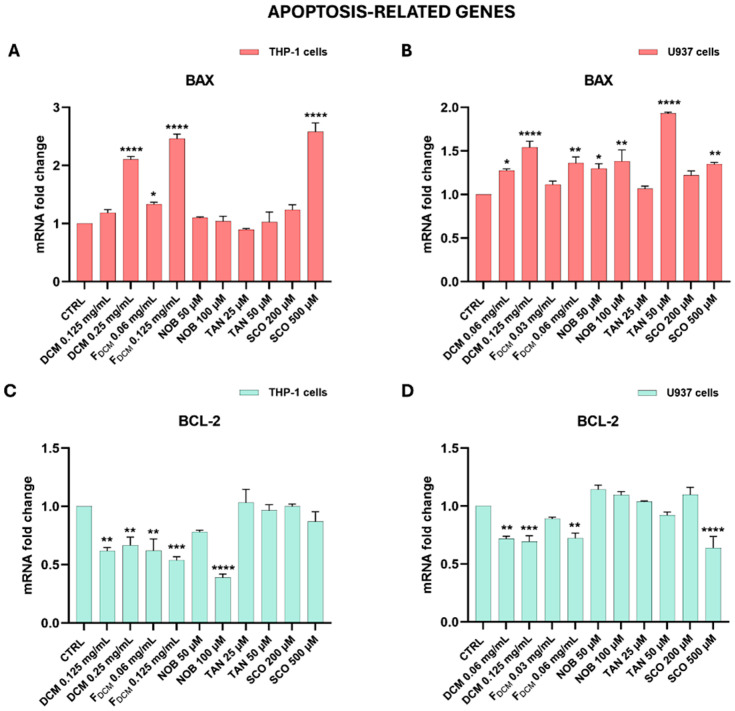
**Expression of apoptosis-related genes in acute myeloid leukemia cells treated with dichloromethane (DCM) extract, combined dichloromethane fractions (F_DCM_), nobiletin (NOB), tangeretin (TAN), and scoparone (SCO).** Leukemia THP-1 cells (**A**,**C**) and U937 cells (**B**,**D**) were treated with DCM extract, F_DCM_, NOB, TAN, and SCO at the indicated concentrations for 24 h before being processed for gene expression analysis. The mRNA levels of *BAX* (light red bars) and *BCL-2* (light green bars) were quantified via RT-PCR using the 2^−ΔΔCT^ method and β-actin as an endogenous control. Results are indicated as *n*-fold change relative to untreated cells (CTRL). Data are expressed as the mean ± SEM of three experiments performed with three replicates (*n* = 9). * *p* <0.05, ** *p* < 0.01, *** *p* < 0.001, and **** *p* < 0.0001 vs. CTRL.

To investigate the mechanism underlying apoptosis, this study evaluated the gene expression of both *BAX* and *BCL-2* in treated AML cells (Figure 11).

Figure 11A shows that the treatment of THP-1 cells with 0.25 mg/mL DCM extract increased the expression of the pro-apoptotic *BAX* gene by 2.11 ± 0.05 fold compared to that in untreated cells (*p* < 0.0001), as well as treatment with F_DCM_, which increased *BAX* expression by 2.46 ± 0.08-fold at the 0.125 mg/mL concentration (*p* < 0.0001). Similarly, 500 µM SCO increased *BAX* gene expression by 2.58 ± 0.15-fold (*p* < 0.0001), while NOB and TAN did not alter it. According to cytofluorimetric data, the gene modulation of *BAX* started at a lower concentration in U937 cells than in THP-1 cells (Figure 11B). Indeed, 0.06 mg/mL DCM extract significantly augmented *BAX* expression by 1.27 ± 0.02-fold (*p* < 0.05) compared to that found in untreated cells and *BAX* expression increased even more with 0.125 mg/mL (by 1.54 ± 0.07-fold; *p* < 0.0001). F_DCM_ at the 0.06 mg/mL concentration caused the same *BAX* expression increase induced by the DCM extract (*p* < 0.05; Figure 11B). Moreover, 50 and 100 µM NOB statistically increased *BAX* expression levels in U937 cells (by between 1.30 and 1.40-fold), similar to 500 µM SCO, whereas 50 µM TAN appeared to be more effective, increasing *BAX* expression by 1.93 ± 0.01-fold vs. CTRL (*p* < 0.0001).

As shown in Figure 11C, the expression of the anti-apoptotic *BCL-2* gene decreased in THP-1 cells treated with the DCM extract (0.125 and 0.25 mg/mL) by about 1.7 ± 0.07-fold compared to that in control cells (*p* < 0.01). Similar results occurred with F_DCM_, and even more with 100 µM NOB (2.56 ± 0.03-fold decrease, *p* < 0.0001). In U937 cells, the DCM extract (0.06 and 0.125 mg/mL) led to a reduction in the expression of *BCL-2* by 1.43 ± 0.05-fold (*p* < 0.01/*p* < 0.001), as well as F_DCM_ at the 0.06 mg/mL concentration (Figure 11D). Finally, only 500 µM SCO induced a significant diminution of *BCL-2* gene expression in treated cells (1.56 ± 0.10-fold decrease, *p* < 0.0001). Conversely, the study did not observe any effect on *BCL-2* expression after treatment with TAN in both cell lines.

### 2.6. Cell Cycle Analysis

To further explore the possible mechanisms responsible for the reduction in cell growth, the cell cycles of treated THP-1 and U937 cells were analyzed.

#### 2.6.1. Both Dichloromethane Extract and Fractions Altered the Cell Cycle of Acute Myeloid Leukemia Cells

**Figure 12:**
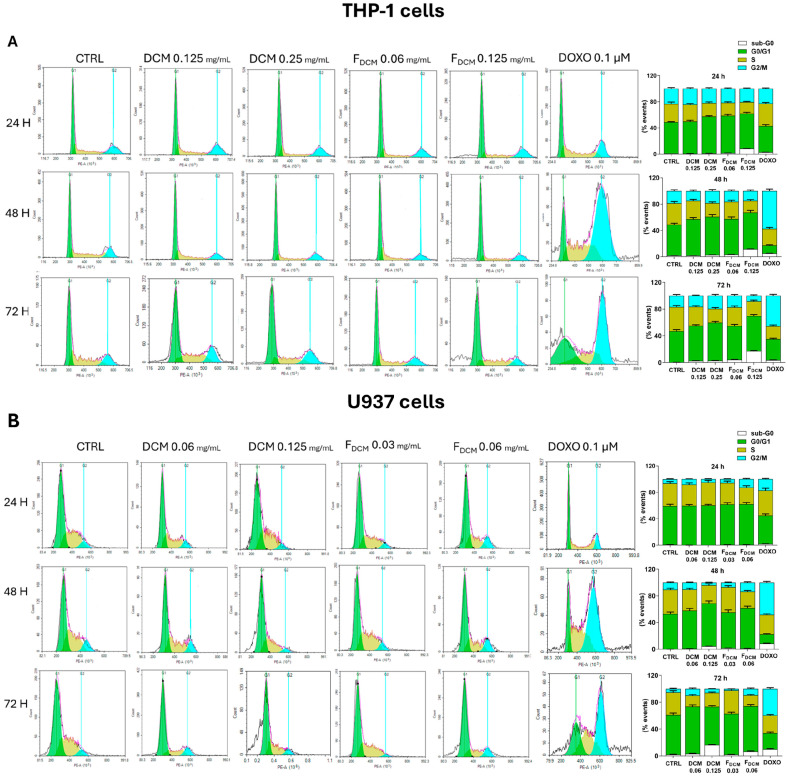
**Influence on cell cycle of acute myeloid leukemia cells exposed to dichloromethane (DCM) extract and fractions (F_DCM_).** THP-1 (**A**) and U937 cells (**B**) were treated with increasing concentrations of DCM extract or F_DCM_ for 24, 48, and 72 h. Doxorubicin was used as a positive control. The distribution of cells through cell cycle phases sub-G0 (white bar), G0/G1 (green bar), S (dark yellow bar), and G2/M (cyan bar) was evaluated using the propidium iodide staining assay. On the left, representative plots are shown. On the right, histograms report the percentage of cells for each phase of the cell cycle, expressed as the mean ± SEM of three independent experiments performed in triplicate (*n* = 9). Vertical lines were set by NovoExpress 1.6.2. software to define the different phases (green line for G0/G1 and light blue for G2/M) based on DNA content. The pink shadow shows the real signal. It matches the black shadow, which shows the signal set by instrument.

The treatment of THP-1 cells with the DCM extract led to a reduction in the number of cells in the S phase due to their accumulation in the G1 phase (Figure 12A; green bar), along with a slight increase in the population in the sub-G0 phase. A similar trend was observed when cells were treated with F_DCM_.

The DCM extract also influenced the cell cycle of U937 cells (Figure 12B), causing a progressive increase in the number of cells in the G1 phase, which was less evident after 72 h of incubation with the 0.125 mg/mL concentration, due to cell accumulation in the sub-G0 phase (16.6 ± 0.5%). Similar results, although to a greater extent, were obtained when cells were treated with F_DCM_.

#### 2.6.2. Cell Cycle Arrest in G1 Phase Induced by Nobiletin, Tangeretin, and Scoparone in Acute Myeloid Leukemia Cells

**Figure 13:**
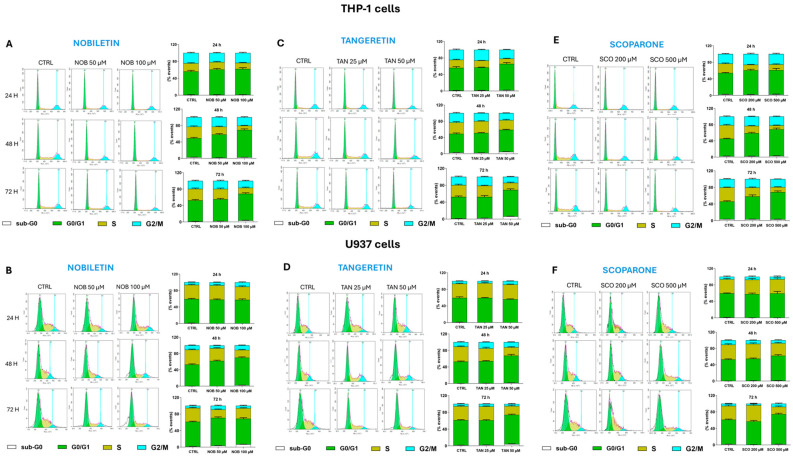
**Cell cycle analysis in acute myeloid leukemia cells treated with nobiletin (NOB), tangeretin (TAN), or scoparone (SCO)**. Progression of THP-1 (**upper side**) and U937 cells (**lower side**) through the cell cycle phases was assessed after cell exposure to NOB (**A**,**B**), TAN (**C**,**D**), and SCO (**E**,**F**) for 24, 48, and 72 h. Representative plots of three independent experimental sessions are shown on the left of each sub-figure, while histograms, on the right side, report the percentage of cells in sub-G0 (white bar), G0/G1 (green bar), S (dark yellow bar), and G2/M (cyan bar) phases. Data are expressed as the mean *±* SEM of three sets of experiments performed in triplicate (*n* = 9). The negative control (CTRL) for THP-1 cells treated with NOB and TAN is the same, so CTRL plots shown in A and C are the same. Similarly, experiments on U937 cells were performed using same negative control, so CTRL plots in B, D, and F are identical. Vertical lines were set by NovoExpress 1.6.2. software to define the different phases (green line for G0/G1 and light blue for G2/M) based on DNA content. The pink shadow shows the real signal. It matches the black shadow, which shows the signal set by instrument.

This study then evaluated whether NOB, TAN, and SCO might alter the progression of AML cells through the cell cycle. Indeed, both THP-1 (Figure 13A) and U937 (Figure 13B) cells treated with 100 µM NOB for 48 h were blocked in the G1 phase (20.3 ± 1.1% and 15 ± 1.8%, respectively), which was maintained after 72 h of incubation, when a sub-G0 population appeared. The effects of TAN on the cell cycle were similar to those induced by NOB in both AML cell lines (Figure 13C,D), whereas SCO appeared to be more effective against THP-1 cells (Figure 13E) than against U937 cells (Figure 13F).

#### 2.6.3. Dichloromethane Extract, Fractions, and Single Compounds Reduced the Gene Expression of Cell Cycle Regulators in Acute Myeloid Leukemia Cells

**Figure 14:**
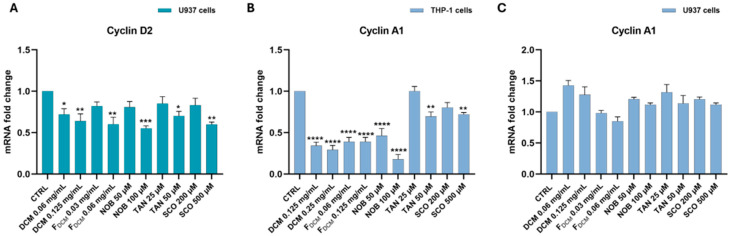
**Gene expression of *CYCLIN A1* and *CYCLIN D2* in acute myeloid leukemia cells treated with dichloromethane (DCM) extract, fractions (F_DCM_), nobiletin (NOB), tangeretin (TAN), and scoparone (SCO).** THP-1 cells or U937 cells were exposed to different concentrations of DCM extract, F_DCM_, NOB, TAN, and SCO for 24 h. The mRNA levels of *CYCLIN D2* (**A**) and *CYCLIN A1* (**B**,**C**) were quantified via RT-PCR using the 2^−ΔΔCT^ method and β-actin as the housekeeping gene. Results are expressed as *n*-fold change relative to untreated cells (control, CTRL). Data are represented as the mean *±* SEM of three different sets of experiments performed in triplicate (*n* = 9). * *p* < 0.05, ** *p* < 0.01, *** *p* < 0.001, and **** *p* < 0.0001 vs. CTRL.

*CYCLIN D2* regulates the G1 phase of the cell cycle and its overexpression in AML is responsible for uncontrolled cell cycle progression and leukemia cell proliferation [21]. In this regard, the study observed that the DCM extract, F_DCM_, and isolated compounds caused a significant reduction in *CYCLIN D2* gene expression in U937 cells (Figure 14A). In particular, exposure to 0.125 mg/mL DCM extract lowered *CYCLIN D2* expression by 1.6 ± 0.1-fold (*p* < 0.01), as occurred with 0.06 mg/mL F_DCM_. Similarly, the single molecules caused a significant downregulation of *CYCLIN D2* expression in U937 cells compared to that in untreated ones (by 1.8 ± 0.03-fold with 100 µM NOB; by 1.4 ± 0.05-fold with 50 µM TAN; by 1.7 ± 0.03-fold with 500 µM SCO). On the contrary, in monocytic THP-1 cells, the study did not detect appreciable levels of *CYCLIN D2*.

Therefore, the study explored the expression levels of *CYCLIN A1*, which is often overexpressed in AML cells, contributing to their proliferation, inhibiting apoptosis, and regulating the G1/S transition [22,23,24]. Interestingly, in THP-1 cells, the DCM extract reduced *CYCLIN A1* expression by about 3 ± 0.04-fold (*p* < 0.0001) at both 0.125 and 0.25 mg/mL compared to that in control cells, similar to F_DCM_ (by 2.55 ± 0.05-fold; *p* < 0.0001). The reduction in *CYCLIN A1* expression also occurred after THP-1 cell treatment with NOB (by 5.56 ± 0.06-fold with 100 µM; *p* < 0.0001), as well as with 50 µM TAN and 500 µM SCO (both by about 1.4-fold) (Figure 14B). However, none of the treatments modified *CYCLIN A1* mRNA levels in U937 cells (Figure 14C).

#### 2.6.4. Dichloromethane Extract, Fractions, and Single Compounds Reduced the Invasiveness of Acute Myeloid Leukemia Cells by Increasing *TIMP-2* Expression

**Figure 15:**
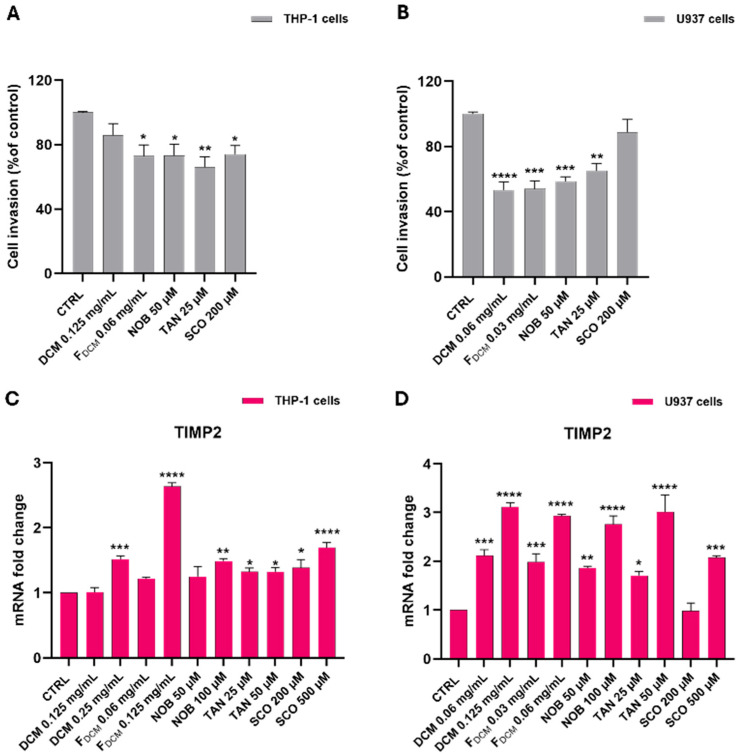
**Dichloromethane (DCM)extract, combined fractions (F_DCM_), nobiletin (NOB), tangeretin (TAN), and scoparone (SCO) reduced the invasiveness of THP-1 and U937 cells by upregulating *TIMP-2* expression**. THP-1 cells (**on the left**) and U937 cells (**on the right**) were exposed to different concentrations of DCM extract, F_DCM_, NOB, TAN, and SCO for 24 h. For the invasion assay (**A**,**B**), leukemia cells migrating through the filter of Matrigel invasion chambers were counted and the invasion rate is expressed as a percentage relative to the control. For RT-PCR analysis (**C**,**D**), the mRNA levels of *TIMP-2* were quantified using the 2^−ΔΔCT^ method and β-actin as the housekeeping gene. Results of qPCR are expressed as *n*-fold change relative to untreated cells (control, CTRL). All data are represented as the mean *±* SEM of three different sets of experiments performed in triplicate (*n* = 9). * *p* < 0.05, ** *p* < 0.01, *** *p* < 0.001, and **** *p* < 0.0001 vs. CTRL.

Further experiments examined the effects of treatments on the invasion capability of AML cells. As shown in Figure 15, treatment with the DCM extract, F_DCM_, and single compounds significantly reduced the invasiveness of AML cells compared to untreated cells (Figure 15A,B).

On this basis, this study investigated the gene expression of *TIMP-2*, which is an inhibitor of cancer invasiveness [25]. The incubation of THP-1 cells with both the DCM extract and F_DCM_ induced a significant increase in *TIMP-2* gene expression compared to that in untreated cells (by 1.52 ± 0.05 fold with 0.25 mg/mL; *p* < 0.001; by 2.64 ± 0.05 fold with 0.125 mg/mL; *p* < 0.0001), as well as exposure to NOB, TAN, and SCO (Figure 15C).

In U937 cells, the treatment with the DCM extract and F_DCM_ yielded similar results: *TIMP-2* expression levels increased by up to 3 ± 0.09-fold compared to that in control cells. Consistently, the treatment of U937 cells with isolated compounds caused a significant upregulation of *TIMP-2* expression (Figure 15D).

## 3. Discussion

The evolution of human beings has driven changes in both food production and consumption over time, and the promotion of healthy eating habits and lifestyles, including eating plant foods such as cereals, vegetables, legumes, olive oil, and citrus fruits. [26]. In recent decades, citrus production and trade have constantly increased and, according to estimates by the Food and Agriculture Organization of the United Nations (FAO), about one-third of citrus fruits produced worldwide are processed [2]. As a result, the massive generation of citrus waste has led to the adoption of more sustainable practices so that “what was earlier considered to be waste becomes a resource”, such as the recovery of added-value compounds from citrus peels [27,28].

Recently, mandarin peel has been demonstrated to possess relevant biological properties, such as anticancer effects, by reducing cell proliferation, migration, and invasion in different models of solid tumors [15,16]. Regarding leukemia, an extract of *C. reticulata* pericarpium, along with its organic fraction, inhibited proliferation and induced the differentiation of a murine cell clone of myeloid leukemia [29]. Moreover, the higher flavonoid content detected in mandarin peel was responsible for its activity against leukemia HL-60 cells, compared to inactive grapefruit and lemon peels [17]. Our study aimed to evaluate the anticancer effects of a *C. reticulata* Blanco peel powder on AML-M5 cells.

The first step of our study consisted of a phytochemical investigation. This study used bioassay-guided isolation since this is a well-established method used to identify biologically active compounds from complex natural mixtures. This process starts with obtaining a crude extract, which is divided into smaller fractions. Each fraction is tested using a bioassay for the desired activity (e.g., for antibacterial, anticancer, or antioxidant effects), and then the active fractions are further separated using chromatographic techniques. Thereby, one or more molecules that are responsible for the activity can be spectroscopically identified, as recently reported [30].

Therefore, the Soxhlet extraction of *C. reticulata* peel led to *n*-hexane and DCM extracts, which, unlike the MeOH extract [31], have not been previously investigated. They were preliminarily screened on TLC plates, and their TLC patterns under UV light suggested the presence of terpenes/coumarins and phenolic compounds, according to what is known about *C. reticulata* peel [27,32]. These same components displayed antioxidant activity, thus developing typical yellow spots in TLC plates sprayed with DPPH radical solution. Moreover, 1D NMR spectroscopic analysis confirmed the abovementioned chemical composition within both the *n*-hexane and DCM extracts. Chemical groups such as aromatic, olefinic, methoxy, and aliphatic groups were related to the molecular structures of flavonoids, including PMFs, and phenolic compounds that were already identified in different varieties of *C. reticulata* peel [32,33].

Once both extracts were characterized, this study used the MTT assay to test their antiproliferative activity in THP-1 and U937 cells, employed as *in vitro* models of AML [34], thus recording significant effects. In addition, the *n*-hexane and DCM extracts showed similar activity profiles against AML cells, which reflected the similarity of their ^1^HNMR spectra. Our findings on the capability of the *n*-hexane extract to reduce AML cell growth are in line with those of Lim and co-workers [35], who showed the antiproliferative effect of an *n*-hexane fraction isolated from *C. grandis* Osbeck fruits in U937 cells. Moreover, Choi et al. [36] documented that an extract of *C. unshiu* peel (known as “satsuma mandarin”) in chloroform, a solvent very similar to DCM, reduced the cell growth of HeLa cancer cells at concentrations similar to those used in this study.

As discussed in the results, further experiments were conducted with only the DCM extract due to its better activity and solubility than the *n*-hexane extract. Therefore, the DCM extract was subjected to chromatographic fractionation via VLC, yielding ten different fractions, which were then screened via TLC. Thereby, the F4 and F5 extract fractions were recognized as the most representative ones (being 68% of the total weight of the extract and developing similar spots on the TLC plate as the progenitor extract), and, since they exhibited similar chemical compositions (fractions eluted using similar mobile phases, i.e., 10% and 20% MeOH in DCM, respectively) and TLC fingerprints, they were combined to give F_DCM_. Then, F_DCM_ was tested in AML cells using the MTT assay. Interestingly, low concentrations of F_DCM_ significantly inhibited the proliferation of THP-1 and U937 cells. This guided the isolation of single compounds. Thus, F_DCM_ was subjected to PTLC, a long-established method of isolation among researchers working in natural products chemistry. Indeed, unlike high-pressure liquid chromatography and counter-current chromatography techniques, PTLC offers advantages. PTLC does not require expensive equipment and needs only minimal training. It allows rapid separation and can isolate natural compounds in the range of 1 mg and 1 g, which is sufficient for structure elucidation purposes. This method can be readily scaled from analytical to preparative formats. During development, the solvent system can be changed easily during a run, and multiple samples can be processed in parallel. These features make PTLC a practical and convenient purification step in natural product isolation [37].

Therefore, the PTLC separation of F_DCM_ resulted in compounds/mixtures A–F, which were further screened via analytical TLC to measure their purity. The purest samples were chemically identified via 1D NMR spectroscopic analysis and literature comparison as the flavonoids nobiletin (A), 3′-demethoxy nobiletin (B or tangeretin), and 6,7-dimethoxycoumarin (D or scoparone).

In line with our findings, different PMFs, including NOB and TAN, were isolated from the peels of *C. reticulata* varieties and studied for their relevant biological activities, including their antiproliferative effects [38]. Of note, the coumarin SCO was previously identified in the peel of another citrus fruit, i.e., *C. trifoliata* (trifoliate orange) [39] and in the *n*-hexane fraction of *C. reticulata* stem bark [40], but not yet in its peel.

Once isolated, the study tested the biological activity of NOB, TAN, and SCO in AML cells using the MTT proliferation assay. Both TAN and NOB reduced both THP-1 and U937 cell growth to a close extent, probably due to their similar chemical structures. Consistently, both TAN and NOB inhibited the proliferation of leukemia HL-60 promyelocytes at micromolar concentrations [41,42] and NOB reduced THP-1 cell growth through downregulation of the c-KIT critical proto-oncogene [43]. Even the coumarin SCO exerted antiproliferative effects in both THP-1 and U937 cells, albeit at long times and high micromolar concentrations. Interestingly, the same range of active concentrations (i.e., 200–500 µM) was found to induce antiproliferative effects in other cancer cell lines [44,45], yet this study is the first to report the leukemia context.

Overall, promising results in AML cells pave the way for further investigations from a pharmacological point of view to define the involved molecular mechanisms and advance current knowledge.

It is known that drugs aiming to restore apoptosis in leukemia cells have shown encouraging results in AML patients [46], as well as in necrotic cancer cells, which may release damage-associated molecular patterns (DAMPs), stimulating immune responses against the tumor [47]. Along this line, this study observed through the Annexin V/propidium iodide assay that the DCM extract, and even more F_DCM_, induced AML cell death through both apoptosis and necrosis, suggesting that different molecules within the extract may drive cells in these two directions. Gene expression studies showed that both the DCM extract and F_DCM_ caused significant modulation of *BAX* and *BCL-2* genes in both THP-1 and U937 cells, suggesting the activation of an apoptotic intrinsic pathway that is directly involved in AML pathogenesis [48]. A similar mechanism of action by a *C. grandis* peel DCM fraction was reported in HepG2 cancer cells [49].

In AML, the cell cycle G1/S transition is altered, prompting cells to continue division and replication in the S phase or leading cells to exit the cell cycle, thus remaining immature and undifferentiated [50]. In other words, blockage of the cell cycle in the G1 phase could limit the entry of leukemia cells into the S replication phase or trigger their apoptosis. In this regard, the increased number of cells in the G1 phase and their reduction in the S phase by both the DCM extract and F_DCM_, as detected via propidium iodide staining, represents another way of inducing apoptosis. The published literature supports our findings, reporting G0/G1 phase blockage followed by apoptosis in lymphoma cells treated with *C. reticulata* peel extract [6]. Moreover, in treated monoblastic U937 cells, this study observed through RT-PCR a significant reduction in the gene expression of *CYCLIN D2*, a known regulator of the G1 phase, which is often upregulated in AML monoblasts, thus promoting uncontrolled cell cycle progression and leukemia proliferation [34,51,52]. This gene reduction did not occur in THP-1 cells, in which the study found a reduction in gene expression of *CYCLIN A1*, another key regulator of the G1 phase implicated in AML-M5 leukemogenesis [22,53]. This finding is likely due to differences between the two cell types. Indeed, the expression levels of *CYCLIN D2* in THP-1 cells, having monocytic features, are poorly detected [34] since they are often induced under cell stimulation/differentiation conditions [54]. Otherwise, U937 cells are characterized by constitutive *CYCLIN A1* expression levels [23], unlike THP-1 cells, which express the highest levels of *CYCLIN A1* among leukemia cells [53]. Cell cycle arrest in the G1 phase linked to the downregulation of *CYCLIN D/E* expression has been reported in multiple cancer cell lines exposed to citrus peel extracts [55]; however, until now, no study correlated *C. reticulata* peel and *CYCLIN A1* expression.

Treatment of either THP-1 or U937 cells with the single isolated compounds i.e., NOB, TAN, and SCO, as analyzed via flow cytometry, resulted in only apoptotic cell death, aligning with previous data on the activity of both NOB and TAN in leukemia HL-60 cells [41,42]. For the first time, this study shows the pro-apoptotic effects of SCO in leukemia cells, although it has been reported in other cancer cell lines [44,56,57]. Therefore, the data indicate that molecules other than NOB, TAN, and SCO present in *C. reticulata* peel may be responsible for the necrotic cell death observed. Indeed, studies have revealed that citrus peels contain a variety of bioactive compounds. These include flavonoids, such as naringin and hesperidin; PMFs, such as NOB, TAN, and sinensetin; phenolic acids, such as ferulic, *p*-coumaric, and caffeic acids; dietary fibers and pectin; essential oils, consisting mostly of D-limonene along with other monoterpenes and oxygenated terpenes; coumarins, such as bergapten, auraptene, and psoralen; limonoids, such as limonin and nomilin derivatives; carotenoids, such as lutein and β-cryptoxanthin; and alkaloids, such as synephrine [58,59]. Of note, NOB and TAN are among the most abundant PMFs identified in the peels of *C. reticulata* cultivars [60,61]. Moreover, although SCO is a coumarin known to be biosynthesized in response to stress stimuli in some citrus peels [62,63], it occurs naturally in the peel of *C. reticulata*, displaying biological potential.

In this regard, this study documented via RT-PCR that high concentrations of SCO regulate the expression of *BAX* and *BCL-2* genes in both THP-1 and U937 cells, as reported in other cancer cells [56], while NOB and TAN modulate these apoptosis-related genes differently in the two AML cell lines. Indeed, it has been reported that TAN and NOB act on other apoptosis regulators such as caspases, MAPKs, or the PI3K/AKT pathway [42,64], thus partially explaining why the study detected limited involvement of *BAX* and *BCL-2* at the gene level in pro-apoptotic actions in both THP-1 and U937 cells. The pro-apoptotic effect can also result from the G0/G1 cell cycle arrest caused by NOB and TAN, which was found in both AML cell lines, as previously suggested for NOB in leukemia HL-60 and THP-1 cells [42,43], whereas this was not previously shown in U937 cells. The SCO-induced THP-1 and U937 cell accumulation in the G0/G1 phase, detected cytofluorimetrically, agrees with what has already been observed in other cancer types [44]. The cell cycle arrest by the compounds employed in our study was mediated at the gene level by reductions in *CYCLIN D2* expression in U937 cells and *CYCLIN A1* expression in THP-1 cells for the same reasons explained above with the DCM extract and F_DCM_. Of note, this study is the first to correlate NOB and SCO with *CYCLIN A1* expression, which has already been correlated with TAN [65]. In addition, it was shown that 80 µM NOB decreased the protein levels of cyclin D2 in leukemia K562 cells [66], and that both TAN and SCO downregulated *CYCLIN D1* expression in non-hematological tumor cell lines [65,67]. Interestingly, a recent study suggested therapies targeting the cell cycle and apoptosis as a strategy to overcome chemotherapy resistance in acute myeloid leukemia [68].

A main feature of monocyte-related AML subtypes (i.e., M4/M5) is the presence of extramedullary infiltrates of leukemia cells in the gingival mucosa, spleen, or skin, which frequently occurs during disease onset [69]. In this regard, it is known that matrix metalloproteinase (MMP) inhibitors are pivotal in the invasiveness of hematological malignancies, including AML-M5 [69,70]; hence, the tissue inhibitor of metalloproteinase-2 (TIMP-2) may play an important role as a suppressor of cancer invasiveness [25]. Interestingly, this study documented through an invasion test that the DCM extract, F_DCM_, as well as NOB, TAN, and SCO reduced the invasiveness of both AML cells and increased their *TIMP-2* gene expression, suggesting their possible role as anti-invasive agents. Therefore, despite its controversial role in leukemia [71], TIMP-2 could inhibit MMP-2 and MMP-9, thus counteracting AML invasiveness [72,73]. Consistently, reduced *TIMP-2* mRNA and protein levels were associated with invasive features in THP-1 cells [71]. Moreover, in other hematological malignancies, such as chronic myeloid leukemia and myeloma multiple, the transcriptional silencing of *TIMP-2* promoted cell proliferation and inhibited apoptosis [74,75], supporting its additional antiproliferative role. This study is the first to describe an increase in *TIMP-2* expression after treatment with *C. reticulata* peel extract, NOB, and SCO, while it has already been shown that TAN enhances *TIMP-2* expression in LPS-stimulated microglial cells [76]. Evidence that NOB and SCO reduce the expression of TIMP-2 downstreamers, such as MMPs, in other cancer cell lines [67,77] further supports these findings.

## 4. Materials and Methods

### 4.1. General Experimental Procedures

Proton (^1^H) NMR spectra (one-dimensional NMR analysis) were recorded using a Bruker AMX Ultrashield NMR spectrometer (Bruker, Billerica, MA, USA) at 600 MHz, with deuterium locking. Chemical shifts are reported in δ (ppm) using CDCl_3_ as an internal standard and coupling constants (*J*) are reported in Hz. Pre-coated silica gel 60 F_254_ aluminum foil (Sigma Aldrich, Darmstadt, Germany) was used to perform analytical thin-layer chromatography (TLC). Vacuum liquid chromatography (VLC) was performed on a column equipped with 60 H silica gel (Sigma-Aldrich, London, UK) under vacuum conditions. All solvents used for extraction, fractionation, and chromatographic analyses were of analytical grade and supplied by Fisher Scientific (Fisher Scientific, Loughborough, Leicestershire, UK) without additional purification. Absorbance measurements for the MTT assay were recorded using a microplate reader (Bio-Rad Laboratories, Milan, Italy). Nobiletin and tangeretin standards were obtained from Sigma-Aldrich and the scoparone standard was obtained from MedChemExpress (MedChemExpress, Monmouth Junction, NJ, USA).

### 4.2. Plant Material Collection

The peels of *Citrus reticulata* Blanco (family: Rutaceae) were obtained from processed fruits (mandarins) obtained from vendors in the United Kingdom in 2023. After collection, samples were carefully cleaned to remove debris, washed with deionized water, and air-dried at room temperature. Finally, the dried peels were ground into a fine powder using a 200-mesh coffee grinder and stored in darkness at the School of Pharmacy and Biomolecular Sciences, Liverpool John Moores University, before their use.

### 4.3. Extraction

The air-dried ground peel (317.03 g) of *C. reticulata* was subjected to Soxhlet extraction with solvents of increasing polarity, such as *n*-hexane, dichloromethane (DCM), and methanol (MeOH), which were added sequentially (900 mL of each solvent for a ten-cycle process at temperatures close to the boiling point of the solvent). All three crude extracts (*n*-hexane, DCM, and MeOH) were then filtered using Whatman^®^ filter paper (Sigma Aldrich, London, UK) and brought to dryness using a rotary evaporator (Cole-Parmer^®^, Altrincham, Cheshire, UK), before being stored at 4 °C.

### 4.4. Fractionation

The DCM extract of *C. reticulata* peel (1.44 g) was separated into different fractions via vacuum liquid chromatography (VLC), according to the method reported by Sarker and Nahar (2012) [78]. In this method, the column was a Büchner funnel equipped with a sintered glass disk and flat filter paper at the bottom. The stationary phase placed into the column consisted of TLC-grade silica gel 60 H (Sigma Aldrich/Merck^®^, Gillingham, Dorset, UK)/GF 254 (Merck^®^, Gillingham, Dorset, UK). Separation was carried out under vacuum conditions in order to obtain homogeneous packing of the column and accelerate the flow rate of the mobile phase. The fractionation (200 mL of each solvent mixture used) included a starting elution with *n*-hexane-DCM mixtures (i.e., 100% *n*-hexane, 50% and 100% DCM in *n*-hexane), then increasing polarity with MeOH, yielding ten fractions (F1–F10). The resulting fractions were evaporated to remove the solvent using a rotary evaporator and stored at 4 °C, before their use in further *in vitro* evaluations.

### 4.5. Isolation of Compounds

Chromatographic techniques such as thin-layer chromatography (TLC) were employed for the screening of crude extracts and the separation of pure compounds from DCM fractions (F_DCM_). Subsequently, spectroscopic techniques such as one-dimensional (1D) NMR were used as tools for elucidating the chemical composition of the crude extracts as well as defining the structure of isolated compounds.

#### 4.5.1. Analytical Thin-Layer Chromatography (TLC)

Analytical thin-layer chromatography (TLC) was carried out on pre-coated silica gel 60 F_254_ plates (0.1–0.2 mm sorbent thickness; Sigma Aldrich, Darmstadt, Germany), with quercetin (1 mg/mL in MeOH) used as a positive control. Lipophilic components were separated using a developing solvent system of 25% ethyl acetate (EtOAc) in *n*-hexane. Spots on developed TLC plates were detected using a UV/vis spectrophotometer (CAMAG Co., Muttenz, Switzerland) at short and long wavelengths of 254 and 365 nm, respectively. TLC plates were sprayed with different reagents to detect different types of compounds.

The developed TLC plate was sprayed with DPPH purple solution (80 µg/mL) using an atomizer and incubated for 30 min. The change in spot color from purple to yellow was due to the reaction between radical DPPH and some antioxidant components, indicating the presence of molecules endowed with free-radical scavenging activity within extracts/fractions.

Another TLC plate was sprayed with p-anisaldehyde-sulfuric acid reagent and heated at 100 °C for 5 min. The latter reagent was used to detect different classes of compounds according to the specific color change, including phenols, sugars, steroids and terpenes.

#### 4.5.2. Preparative Thin-Layer Chromatography (PTLC)

Preparative TLC (PTLC) was performed according to the standard protocol by Gibbon and co-workers [37] to further purify F_DCM_ and isolate related compounds. PTLC is an adsorption chromatography where the sorbent is silica. Analytical TLC analyses were conducted to find the optimized solvent system for PTLC work. Then, thin lines (2–4 mm thick) of F_DCM_ (10 mg/mL in DCM) were applied to the bottom of the silica TLC plate (200 × 200 × 0.25 mm and 0.5–4 mm sorbent thickness; Merck^®^) using a thin capillary. Application of the sample as a straight line (by drawing a pencil line 1.5 cm above the plate edge) was necessary to ensure separation of the sample into uniform bands of compounds. Subsequently, the TLC plate was placed into a glass tank containing the solvent (a solvent system of 25% EtOAc in 100 mL *n*-hexane), which wet the part below where the line was applied. A solvent-saturated atmosphere was produced by adding a clean filter paper (15 × 15 cm) in the developing tank to improve chromatographic separation. The solvent migrated up the plate due to capillary action (development phase). Finally, the plate was removed from the tank and air-dried in a fume cupboard.

The effectiveness of separation depends on the complexity of the extract (i.e., DCM extract), which therefore was partially purified via VLC (into F_DCM_) prior to being subjected to PTLC. In addition, multiple developments (by running at least 5 plates) were necessary to obtain a quantitative separation of each component. Polar compounds, having high affinity for silica (i.e., stationary phase), were retained by the silica sorbent and moved slowly up the plate when the solvent (mobile phase) migrated. On the contrary, nonpolar compounds, having less affinity for silica, moved quickly up the plate. As a result of development, 6 samples (namely A–F) were separated from the F_DCM_ baseline based on their different polarities. Since the separated compounds absorbed UV light at short or long wavelengths, multiple developments were monitored by reading the TLC plates each time under UV light. Finally, six samples were recovered by scraping each separated band off the plate into a foil and then desorbing the compounds from the silica. Desorption consisted of putting each compound rich in silica in a conical flask and adding DCM solvent for 30 min, which facilitated the dissolution of the compound not of silica. The resulting suspension was then filtered to remove the silica. Desorption was repeated three times to ensure a good recovery of the isolated samples. Single samples were dried using a pure N_2_ blow-down apparatus. Finally, the samples were screened under UV light via analytical TLC to ascertain their purity, using analytical plates with a smaller particle size suitable for measuring purity than PTLC plates. The resulting pure compounds were subjected to one-dimensional (1D) nuclear magnetic resonance (NMR) analyses for their structure elucidation.

#### 4.5.3. One-Dimensional NMR

A few milligrams of the *n*-hexane extract (i.e., 13 mg) and DCM extract (i.e., 20.8 mg) were dissolved in 1 mL of deuterated chloroform (CDCl_3_) before being subjected to NMR analysis. The isolated compounds were also dissolved in CDCl_3_. All samples were subjected to NMR analysis at 600 MHz using a Bruker AMX Ultrashield NMR spectrometer (Bruker, Billerica, MA, USA). Wilmad^®^ NMR tubes (OD: 5 mm, length: 7 inches, glass A material; Sigma-Aldrich) and NMR solvents supplied by Cambridge Isotope Laboratories (Cambridge Isotope Laboratories Inc., Andover, MA, USA) were employed for NMR analysis. The one-dimensional NMR analysis included ^1^H NMR spectroscopy, where chemical shifts and couplings are reported on the same axis as frequency. The integration of peaks provided an indication of the number of protons present in each isolated compound, the chemical shift (0–10 ppm) indicated the electronic and chemical environment of each proton, and the spin–spin coupling provided information on the neighboring protons.

### 4.6. Cell Cultures

The human monocytic leukemia THP-1 cell line (originally obtained from ATCC, Rockville, MD, USA) and human monoblastic leukemia U937 cell line (Istituto Zooprofilattico, Brescia, Italy) were used for experiments as *in vitro* AML models. U937 cells were maintained in RPMI-1640 medium enriched with 10% (*v*/*v*) fetal bovine serum, 1 mM sodium pyruvate, 2 mM L-glutamine, 100 U/mL penicillin, and 100 µg/mL streptomycin. For THP-1 cells, the same medium was used with the addition of 10 mM HEPES, 1 mM glucose, and 0.05 mM 2-mercaptoethanol (Sigma-Aldrich). All cell culture reagents were supplied by Euroclone (Milan, Italy). Cells were kept in an incubator at 37 °C under a 5% CO_2_/95% air atmosphere and split into subcultures twice per week to a density of 1–3 × 10^5^ cells/mL [9,34].

### 4.7. Proliferation Assay

The antiproliferative effects of crude extracts, combined DCM fractions (F_DCM_), and single compounds were assessed using the 3-(4,5-dimethylthiazole-2-yl)-2,5-diphenyltetrazolium bromide (MTT) colorimetric assay, as previously reported [79]. Briefly, cells were seeded onto 96-well plates (3 × 10^5^ cells/mL) and exposed to fresh medium (for untreated cells) or medium supplemented with different concentrations of crude extracts (0.008 or 0.06–1 mg/mL), F_DCM_ (0.03–1 mg/mL), TAN (1–50 µM), NOB (1–100 µM), and SCO (1–500 µM) for 24, 48, and 72 h. At the end of the incubation period, plates were centrifuged to remove the treatments and re-incubated with a solution of 0.5 mg/mL MTT (Sigma-Aldrich) for 4 h at 37 °C. The resulting formazan crystals were dissolved by adding 100 µL of 0.1 N HCl/isopropanol lysis solution to each well. The absorbance values were determined using a microplate reader (Bio-Rad Laboratories, Milan, Italy) at 570 nm (reference at 690 nm). Results are shown as the absorbance percentage detected in viable cells relative to untreated cells (control, CTRL). Values of the half-maximal inhibitory concentration (IC_50_) were calculated using GraphPAD Prism Software (version 8.4.2, San Diego, CA, USA) with the log(inhibitor) vs. response—variable slope model.

### 4.8. Cytofluorimetric Analyses

Fluorescence-activated cell sorting (FACS) analysis was carried out to detect apoptotic cell death (by Annexin V/propidium iodide staining assay) and to assess the cell cycle (by propidium iodide staining) in leukemia cell lines exposed to treatments using a Novocyte 2000 flow cytometer (ACEA Biosciences Inc., San Diego, CA, USA).

#### 4.8.1. Apoptosis Evaluation

The Annexin V/propidium iodide staining assay served to discriminate cells in early apoptosis from cells in late apoptosis or necrosis. Indeed, cells undergoing apoptosis bind annexin V with high affinity. Conversely, cells in late apoptosis and necrosis lose their membrane integrity, thus becoming permeable to propidium iodide. Apoptosis evaluation was performed as described by Ferlazzo et al. [80]. Therefore, THP-1 and U937 cells were seeded onto 6-well plates (2 × 10^5^ cells/mL) and treated with DCM extract (0.06, 0.125, 0.25 mg/mL), F_DCM_ (0.03, 0.06, and 0.125 mg/mL), TAN (25 and 50 µM), NOB (50 and 100 µM), and SCO (200 and 500 µM) for 24, 48, and 72 h. At the end of the treatment time, cells were harvested, washed with PBS, and suspended in 200 µL 1× binding buffer and 5 µL Annexin V–fluorescein isothiocyanate (FITC) conjugate, according to the manufacturer’s protocol (BD Biosciences, Milan, Italy). Each sample was gently vortexed and incubated at room temperature for 15 min in the dark. Subsequently, cells were washed with PBS and suspended in 190 µL 1× binding buffer and 10 µL propidium iodide (PI, 20 µg/mL) for analysis.

#### 4.8.2. Cell Cycle Assessment

The propidium iodide staining assay served to visualize the cell distribution among phases of the cell cycle. PI stoichiometrically binds DNA, thus differentiating phases according to their DNA amount. In this assay, THP-1 and U937 cells were seeded onto 6-well plates (2 × 10^5^ cells/mL) and treated with DCM extract, F_DCM_, TAN, NOB, and SCO for 24, 48, and 72 h. At the end of the treatments, cells were processed as previously reported [81]. Briefly, leukemia cells were collected, washed with PBS, and fixed in 70% ice-cold ethanol for at least 2 h at 4° C. Then, cells were washed with PBS and resuspended in 250 µL PBS and 5 µL of ribonuclease A (10 mg/mL; Sigma-Aldrich) for 1 h at 37 °C. Afterward, cells were stained with 10 µL PI (1 mg/mL) and subjected to analysis.

### 4.9. Gene Expression Studies

Gene expressions of *BAX*, *BCL-2*, *CYCLIN A1*, *CYCLIN D2*, and *TIMP-2* were determined via real-time PCR as previously reported [82]. THP-1 and U937 cells were plated into 100 mm Petri dishes at a minimum density of 1 × 10^6^ cells/dish and exposed to fresh medium (for untreated cells) or treatments with increasing concentrations of DCM extract, F_DCM_, TAN, NOB, and SCO for 24 h at 37 °C. RNA was extracted using TRIzol reagent (Invitrogen, Carlsbad, CA, USA) according to the instructions of the producer. An equal amount of RNA (2 µg) from each sample was converted into cDNA using the High-Capacity cDNA Archive Kit (Applied Biosystems, Life Technologies, Foster City, CA, USA). Quantitative PCR was performed on 96-well plates using the 7500 qPCR System (Applied Biosystems) in a reaction volume of 20 µL, according to the following protocol: one cycle at 50 °C for 2 min and 95 °C for 2 min, followed by 45 cycles at 95 °C for 15 s and 60 °C for 1 min. All data were analyzed using the 2^−ΔΔCT^ quantification method against β-actin (*ACTB*), which was used as the housekeeping gene. Values are shown as *n*-fold change compared to untreated cells. The primer sequences employed in the quantitative PCR reaction are summarized in Table 7.

### 4.10. Invasion Assay

The invasion assay was performed using Matrigel invasion chambers, which constituted 24 well-plates equipped with filters of 8 µm pore size coated with Matrigel (Corning Inc., Painted Post, NY, USA), as previously described [71,83]. Therefore, THP-1 or U937 cells (2 × 10^5^ cells/well) were suspended in serum-free RPMI 1640 medium and seeded in the upper compartment of the chamber, while medium supplemented with 10% FBS was added to the lower compartment. After 24 h of incubation at 37 °C with DCM extract, F_DCM_, NOB, TAN, and SCO, leukemia cells that invaded the lower side of the Matrigel-coated filter were harvested and counted via Tripan blue staining using a Neubauer hemocytometer. The invasion rate was calculated from the ratio of the number of cells in the lower compartment compared to the total number of leukemia cells loaded in the upper compartment and expressed as a percentage.

### 4.11. Statistical Analysis

All results are expressed as the mean ± standard error of the mean (SEM) of different replicates according to the assay. Statistical analysis was carried out using one-way analysis of variance (ANOVA), followed by Dunnett’s multiple comparison test (GraphPad Prism Software, version 8.4.2, San Diego, CA, USA). A *p*-value lower or equal to 0.05 (*) was considered statistically significant.

## 5. Conclusions

Mandarin peel is one of the main byproducts of the agrifood industry and continues to attract interest for its biological activities. This study combined a phytochemical investigation with a pharmacological evaluation to characterize the activity of *C. reticulata* peel. A bioassay-guided approach enabled the isolation of the flavonoids nobiletin and tangeretin and the coumarin scoparone, which were structurally confirmed using standard spectroscopic techniques. The findings demonstrate that components of *C. reticulata* peel exerted antiproliferative effects in pediatric and adult AML *in vitro* models through cell cycle arrest and induction of apoptosis, with or without necrosis. For the first time, tangeretin was shown to inhibit the growth of THP-1 and U937 cells, nobiletin induced cell cycle arrest and apoptosis in U937 cells, and scoparone displayed antileukemic activity. This study also provides the first evidence that the extract, its fractions, and the isolated compounds reduced AML cell invasiveness and increased *TIMP-2* expression. Overall, these results support the valorization of mandarin peel as a source of bioactive molecules with potential relevance in the pharmaceutical field. The efficient use of citrus waste, with maximal recovery of valuable compounds and minimal environmental impact, remains an important objective, and this study moves in that direction.

## Figures and Tables

**Table 1:** Percentage yields of *n*-hexane, dichloromethane (DCM), and methanol (MeoH) extracts obtained from *C. reticulata* peel.

Crude Extract	Weight (g)	% Yield
*n*-hexane	3.63	1.14
DCM	1.7	0.54
MeOH	116.74	36.82

**Table 2:** IC_50_ values (mg/mL) of dichloromethane and *n*-hexane extracts from *C. reticulata* peel calculated at 72 h in treated leukemia cells.

Cell Line	IC_50_ Values (mg/mL) of *C. reticulata* Peel Extracts	
	DCM Extract	*n*-Hexane Extract
THP-1	0.27 ± 0.06	0.32 ± 0.07
U937	0.11 ± 0.02	0.20 ± 0.02

**Table 3:** Weights of dichloromethane fractions obtained via vacuum liquid chromatography and related yields.

DCM Fraction	Mobile Phase Gradient	Weight of Fraction (mg)	Yield (%)
F1	100% *n*-hexane	8.6	0.60
F2	50% DCM in *n*-hexane	53.5	3.71
F3	100% DCM	18	1.25
F4	10% MeOH in DCM	980 ^1^	68.05
F5	20% MeOH in DCM		
F6	30% MeOH in DCM	108.3	7.52
F7	40% MeOH in DCM	66.6	4.62
F8	50% MeOH in DCM	43.4	3.01
F9	60% MeOH in DCM	20.7	1.44
F10	70% MeOH in DCM	13.3	0.92

^1^ The richest DCM fractions (i.e., F4 and F5) with similar TLC fingerprints were mixed in their liquid form and then dried, thus yielding a final weight of 980 mg.

**Table 4:** Comparison between IC_50_ values (mg/mL) of Dichloromethane extract and combined fractions in leukemia cells treated for 72 h.

Cell Line	IC_50_ Values (mg/mL)	
	DCM Extract	F_DCM_
THP-1	0.27 ± 0.06	0.15 ± 0.05
U937	0.11 ± 0.02	0.07 ± 0.03

**Table 5:** Weight and yields of samples separated from combined dichloromethane fractions (F_DCM_).

Compounds/Mixtures of Compounds	Weight (mg)	Yield (%)
F4 + F5 baseline	11.1	-
A	0.7	3.4
B	0.5	2.4
C	2	9.8
D	0.5	2.4
E	1.4	6.8
F	4.3	21

**Table 6:** IC_50_ values (µM) of nobiletin, scoparone, and tangeretin in acute myeloid leukemia cells treated for 72 h.

Cell Line	IC_50_ Values (µM)		
	Nobiletin	Tangeretin	Scoparone
THP-1	>100	86.4 ± 1.05	367.9 ± 1.6
U937	48.5 ± 1.4	28.7 ± 1	>500

**Table 7:** Primer sequences used for real-time PCR.

Gene	NCBI Reference Sequence	Primer Sequence
*BAX*	NM_138764.5	Forward: 5′-GGACGAACTGGACAGTAACATGG-3′ Reverse: 5′-GCAAAGTAGAAAAGGGCGACAAC-3′
*BCL-2*	NM_000657.3	Forward: 5′-ATCGCCCTGTGGATGACTGAG-3′ Reverse: 5′-CAGCCAGGAGAAATCAAACAGAGG-3′
*CCND2 (CYCLIN D2)*	NM_001759.4	Forward: 5′- CTGGCCTCCAAACTCAAAGA-3′ Reverse: 5′-TTCCACTTCAACTTCCCCA-3′
*CCNA1 (CYCLIN A1)*	NM_001413923.1	Forward: 5′- CATGAAGAAGCAGCCAGACA-3′ Reverse: 5′- TTCGAAGCCAAAAGCATAGC-3′
*TIMP-2*	NM_003255.5	Forward: 5′-CACCAGGCCAAGTTCTTC-3′ Reverse: 5′-CGGTACCACGCACAGGA -3′
*ACTB*	NM_001101.5	Forward: 5′-TTGTTACAGGAAGTCCCTTGCC-3′ Reverse: 5′-ATGCTATCACCTCCCCTGTGTG-3′

## Data Availability

The original contributions presented in this study are included in the article/Supplementary Materials. Further inquiries can be directed at the corresponding author.

## Supplementary Materials

The following supporting information can be downloaded at: https://www.mdpi.com/article/10.3390/ijms27031256/s1.

## Abbreviations

The following abbreviations are used in this manuscript:

AML	Acute myeloid leukemia
BAX	Bcl-2 associated X-protein
BCL-2	B-cell lymphoma 2
CTRL	Control
DCM	Dichloromethane
DOXO	Doxorubicin
DPPH	2,2-diphenyl-1-picrylhydrazyl
EtOAc	Ethyl acetate
F_DCM_	Combined F4 and F5 dichloromethane fractions
MeOH	Methanol
MTT	3-(4,5-dimethylthiazole-2-yl)-2,5-diphenyltetrazolium bromide
NOB	Nobiletin
PI	Propidium iodide
PMFs	Polymethoxyflavones
PTLC	Preparative thin-layer chromatography
QUE	Quercetin
SCO	Scoparone
TAN	Tangeretin
TIMP-2	Tissue inhibitor of metalloproteinase 2
TLC	Thin-layer chromatography
VLC	Vacuum liquid chromatography

## References

[1] Ramon-Laca L. (2003). The introduction of cultivated citrus to Europe via Northern Africa and the Iberian Peninsula. Econ. Bot..

[2] Food and Agricolture Organization of the United Nations (FAO) (2021). Citrus Fruit Fresh and Processed Statistical Bulletin 2020.

[3] Mabberley D.J. (1997). A classification for edible Citrus (*Rutaceae*). Telopea.

[4] Ho S.C., Lin C.C. (2008). Investigation of heat treating conditions for enhancing the anti-inflammatory activity of citrus fruit (*Citrus reticulata*) peels. J. Agric. Food Chem..

[5] Shorbagi M., Fayek N.M., Shao P., Farag M.A. (2022). *Citrus reticulata* Blanco (the common mandarin) fruit: An updated review of its bioactive, extraction types, food quality, therapeutic merits, and bio-waste valorization practices to maximize its economic value. Food Biosci..

[6] Nair S.A., Sr R.K., Nair A.S., Baby S. (2018). Citrus peels prevent cancer. Phytomedicine.

[7] Sung H., Ferlay J., Siegel R.L., Laversanne M., Soerjomataram I., Jemal A., Bray F. (2021). Global Cancer Statistics 2020: GLOBOCAN Estimates of Incidence and Mortality Worldwide for 36 Cancers in 185 Countries. CA Cancer J. Clin..

[8] Bennett J.M., Catovsky D., Daniel M.T., Flandrin G., Galton D.A., Gralnick H.R., Sultan C. (1976). Proposals for the classification of the acute leukaemias French-American-British (FAB) co-operative group. Br. J. Haematol..

[9] Maugeri A., Russo C., Musumeci L., Lombardo G.E., De Sarro G., Barreca D., Cirmi S., Navarra M. (2022). The Anticancer Effect of a Flavonoid-Rich Extract of Bergamot Juice in THP-1 Cells Engages the SIRT2/AKT/p53 Pathway. Pharmaceutics.

[10] Musumeci L., Maugeri A., Russo C., Lombardo G.E., Cirmi S., Navarra M. (2023). Citrus Flavonoids and Autoimmune Diseases: A Systematic Review of Clinical Studies. Curr. Med. Chem..

[11] Navarra M., Femia A.P., Romagnoli A., Tortora K., Luceri C., Cirmi S., Ferlazzo N., Caderni G. (2020). A flavonoid-rich extract from bergamot juice prevents carcinogenesis in a genetic model of colorectal cancer, the Pirc rat (F344/NTac-Apc(am1137)). Eur. J. Nutr..

[12] Cirmi S., Maugeri A., Lombardo G.E., Russo C., Musumeci L., Gangemi S., Calapai G., Barreca D., Navarra M. (2021). A Flavonoid-Rich Extract of Mandarin Juice Counteracts 6-OHDA-Induced Oxidative Stress in SH-SY5Y Cells and Modulates Parkinson-Related Genes. Antioxidants.

[13] Celano M., Maggisano V., De Rose R.F., Bulotta S., Maiuolo J., Navarra M., Russo D. (2015). Flavonoid Fraction of *Citrus reticulata* Juice Reduces Proliferation and Migration of Anaplastic Thyroid Carcinoma Cells. Nutr. Cancer.

[14] Castro M.A., Rodenak-Kladniew B., Massone A., Polo M., Garcia de Bravo M., Crespo R. (2018). *Citrus reticulata* peel oil inhibits non-small cell lung cancer cell proliferation in culture and implanted in nude mice. Food Funct..

[15] Wen S., Sun L., An R., Zhang W., Xiang L., Li Q., Lai X., Huo M., Li D., Sun S. (2020). A combination of *Citrus reticulata* peel and black tea inhibits migration and invasion of liver cancer via PI3K/AKT and MMPs signaling pathway. Mol. Biol. Rep..

[16] Lee J., Lee J., Kim M., Kim J.H. (2018). Fermented Extraction of *Citrus unshiu* Peel Inhibits Viability and Migration of Human Pancreatic Cancers. J. Med. Food.

[17] Diab K.A. (2016). In Vitro studies on phytochemical content, antioxidant, anticancer, immunomodulatory, and antigenotoxic activities of lemon, grapefruit, and mandarin citrus peels. Asian Pac. J. Cancer Prev..

[18] Sak K., Everaus H. (2017). Established Human Cell Lines as Models to Study Anti-leukemic Effects of Flavonoids. Curr. Genom..

[19] Hamdan D.I., El-Shiekh R.A., El-Sayed M.A., Khalil H.M.A., Mousa M.R., Al-Gendy A.A., El-Shazly A.M. (2020). Phytochemical characterization and anti-inflammatory potential of Egyptian Murcott mandarin cultivar waste (stem, leaves and peel). Food Funct..

[20] Chemical Book. https://www.chemicalbook.com/SpectrumEN_120-08-1_1HNMR.htm.

[21] McKenzie L., Martinez-Soria N., Draper J., Nakjang S., Blair H.J., Wichmann C., Vormoor J., Bonifer C., Lacaud G., Heidenreich O. (2016). Identification of CCND2 as a RUNX1/ETO target Required for leukaemic propagation. Blood.

[22] Fan Y., Hu Y., Yan C., Chen Q., Nguyen C., Dunn B.K., Ries R.E., Bolouri H., Smith J.L., Kolb E.A. (2020). Altered transcriptome in pediatric AML compared with normal hematopoiesis. Br. J. Cancer Res..

[23] Leung W.K., Workineh A., Mukhi S., Tzannou I., Brenner D., Watanabe N., Leen A.M., Lulla P. (2020). Evaluation of cyclin A1-specific T cells as a potential treatment for acute myeloid leukemia. Blood Adv..

[24] Yang X., Wan M., Yu F., Wu X. (2021). Histone methyltransferase EZH2 epigenetically affects CCNA1 expression in acute myeloid leukemia. Cell Signal.

[25] Peeney D., Jensen S.M., Castro N.P., Kumar S., Noonan S., Handler C., Kuznetsov A., Shih J., Tran A.D., Salomon D.S. (2020). TIMP-2 suppresses tumor growth and metastasis in murine model of triple-negative breast cancer. Carcinogenesis.

[26] Miguel G. (2016). Citrus as a component of the Mediterranean Diet. J. Spat. Organ. Dynamics Food Syst. Sustain..

[27] Ferreira S.S., Silva A.M., Nunes F.M. (2018). *Citrus reticulata* Blanco peels as a source of antioxidant and anti-proliferative phenolic compounds. Ind. Crops Prod..

[28] Russo C., Lombardo G.E., Bruschetta G., Rapisarda A., Maugeri A., Navarra M. (2024). Bergamot Byproducts: A Sustainable Source to Counteract Inflammation. Nutrients.

[29] Mak N.K., Wong-Leung Y.L., Chan S.C., Wen J., Leung K.N., Fung M.C. (1996). Isolation of anti-leukemia compounds from *Citrus reticulata*. Life Sci..

[30] Khuniad C., Nahar L., Talukdar A.D., Nath R., Ritchie K.J., Sarker S.D. (2025). Cancer Chemopreventive Potential of *Claoxylon longifolium* Grown in Southern Thailand: A Bioassay-Guided Isolation of Vicenin 1 as the Active Compound and In Silico Studies on Related C-Glycosyl Flavones. Molecules.

[31] Gbaj M.A., Sadawe I.A., Meiqal N.M., Bensaber S.M., Maamar M.S., Hermann A., Gbaj A.M. (2019). Evaluation of neuropharmacological activities of methanolic and aqueous extracts of *Citrus reticulata* (*Rutaceae*) fruit peels. Am. J. Biomed. Sci. Res..

[32] Liang Q.W., Zhan Y.Y., Peng M.L., He P.W., Gadetskaya A.V., Yu K.J., Zhang J., Huang Z.H., Xu W. (2025). Optimized extraction of polymethoxyflavonoids from *Citrus reticulate* ‘Chachi’peel and their anti-atherosclerotic potential. J. Funct. Foods.

[33] Kaur S., Singh V., Chopra H.K., Panesar P.S. (2024). Extraction and characterization of phenolic compounds from mandarin peels using conventional and green techniques: A comparative study. Discov. Food.

[34] Wu P.S., Yen J.H., Chen P.Y., Wu M.J. (2025). Molecular Mechanisms of Biochanin A in AML Cells: Apoptosis Induction and Pathway-Specific Regulation in U937 and THP-1. Int. J. Mol. Sci..

[35] Lim H.-K., Moon J.Y., Kim H., Cho M., Cho S.K. (2009). Induction of apoptosis in U937 human leukaemia cells by the hexane fraction of an extract of immature *Citrus grandis* Osbeck fruits. Food Chem..

[36] Choi Y.S., Han J.M., Kang Y.J., Jung H.J. (2021). Chloroform extract of *Citrus unshiu* Markovich peel induces apoptosis and inhibits stemness in HeLa human cervical cancer cells. Mol. Med. Rep..

[37] Gibbons S. (2012). An introduction to planar chromatography and its application to natural products isolation. Natural Products Isolation.

[38] Duan L., Dou L.L., Yu K.Y., Guo L., Bai-Zhong C., Li P., Liu E.H. (2017). Polymethoxyflavones in peel of *Citrus reticulata* ‘Chachi’ and their biological activities. Food Chem..

[39] Kerekes D., Horvath A., Kusz N., Borcsa B.L., Szemeredi N., Spengler G., Csupor D. (2022). Coumarins, furocoumarins and limonoids of *Citrus trifoliata* and their effects on human colon adenocarcinoma cell lines. Heliyon.

[40] Tahsin T., Wansi J.D., Al-Groshi A., Evans A., Nahar L., Martin C., Sarker S.D. (2017). Cytotoxic Properties of the Stem Bark of *Citrus reticulata* Blanco (*Rutaceae*). Phytother. Res..

[41] Hirano T., Abe K., Gotoh M., Oka K. (1995). Citrus flavone tangeretin inhibits leukaemic HL-60 cell growth partially through induction of apoptosis with less cytotoxicity on normal lymphocytes. Br. J. Cancer.

[42] Hsiao P.C., Lee W.J., Yang S.F., Tan P., Chen H.Y., Lee L.M., Chang J.L., Lai G.M., Chow J.M., Chien M.H. (2014). Nobiletin suppresses the proliferation and induces apoptosis involving MAPKs and caspase-8/-9/-3 signals in human acute myeloid leukemia cells. Tumour Biol..

[43] Chen P.Y., Chen Y.T., Gao W.Y., Wu M.J., Yen J.H. (2018). Nobiletin Down-Regulates c-KIT Gene Expression and Exerts Antileukemic Effects on Human Acute Myeloid Leukemia Cells. J. Agric. Food Chem..

[44] Li N., Yang F., Liu D.Y., Guo J.T., Ge N., Sun S.Y. (2021). Scoparone inhibits pancreatic cancer through PI3K/Akt signaling pathway. World J. Gastrointest. Oncol..

[45] Huang S., Lin L., Ma Y., Zhu Q., Weng N. (2023). Scoparone induces autophagic cell death via the PAK1/AKT axis in colorectal cancer. Eur. J. Pharmacol..

[46] Cassier P.A., Castets M., Belhabri A., Vey N. (2017). Targeting apoptosis in acute myeloid leukaemia. Br. J. Cancer.

[47] Gong Y., Fan Z., Luo G., Yang C., Huang Q., Fan K., Cheng H., Jin K., Ni Q., Yu X. (2019). The role of necroptosis in cancer biology and therapy. Mol. Cancer.

[48] Wei Y., Cao Y., Sun R., Cheng L., Xiong X., Jin X., He X., Lu W., Zhao M. (2020). Targeting Bcl-2 Proteins in Acute Myeloid Leukemia. Front. Oncol..

[49] Eom T., Choi J.H., Kim J., Kim J., Unno T. (2022). Dichloromethane fraction of *Citrus grandis* induces apoptosis in a human colorectal cancer cell lines via apoptotic signaling pathway. J. Funct. Foods.

[50] Schnerch D., Yalcintepe J., Schmidts A., Becker H., Follo M., Engelhardt M., Wäsch R. (2012). Cell cycle control in acute myeloid leukemia. Am. J. Cancer Res..

[51] Fang X., Chen C., Xia F., Yu Z., Zhang Y., Zhang F., Gu H., Wan J., Zhang X., Weng W. (2016). CD274 promotes cell cycle entry of leukemia-initiating cells through JNK/Cyclin D2 signaling. J. Hematol. Oncol..

[52] Mou J., Huang Q., Liu X., Liu W., Liu Y., Mei Y., Gu R., Xu Y., Tang K., Tian Z. (2025). AML1-ETO and CCND2 overexpression cooperate to drive acute myeloid leukemia initiation and progression. J. Leukoc. Biol..

[53] Ochsenreither S., Majeti R., Schmitt T., Stirewalt D., Keilholz U., Loeb K.R., Wood B., Choi Y.E., Bleakley M., Warren E.H. (2012). Cyclin-A1 represents a new immunogenic targetable antigen expressed in acute myeloid leukemia stem cells with characteristics of a cancer-testis antigen. Blood J. Am. Soc. Hematol..

[54] Osei Kuffour E., Schott K., Jaguva Vasudevan A.A., Holler J., Schulz W.A., Lang P.A., Lang K.S., Kim B., Häussinger D., König R. (2018). USP18 (UBP43) abrogates p21-mediated inhibition of HIV-1. J. Virol..

[55] Koolaji N., Shammugasamy B., Schindeler A., Dong Q., Dehghani F., Valtchev P. (2020). Citrus Peel Flavonoids as Potential Cancer Prevention Agents. Curr. Dev. Nutr..

[56] Zheng B., Song W., Liu C., Kou X., Yu Y., Wang Y., Ma J., Liu Y., Jiang J., Xue Z. (2025). Scoparone from *Artemisia capillaris* Thunb. induces apoptosis in HepG2 cells via activation of both intracellular and extracellular pathways. Nat. Prod. Res..

[57] Shen H., Wei Y., Yang Q., Cai Y., Zhu K., Chen X. (2023). Scoparone induces both apoptosis and ferroptosis via multiple mechanisms in non-small-cell lung cancer cells. Toxicol. *Vitr.*.

[58] Sanli I., Ozkan G., Şahin-Yeşilçubuk N. (2025). Green extractions of bioactive compounds from citrus peels and their applications in the food industry. Food Res. Int..

[59] Song Z., Xu J., Tian J., Deng J., Deng X., Peng M., Luo W., Wei M., Li Y., Zheng G. (2025). Differentiating Tangerine Peels from Other *Citrus reticulata* through GC-MS, UPLC-Q-Exactive Orbitrap-MS, and HPLC-PDA. ACS Omega.

[60] Chen Q., Wang D., Tan C., Hu Y., Sundararajan B., Zhou Z. (2020). Profiling of flavonoid and antioxidant activity of fruit tissues from 27 Chinese local citrus cultivars. Plants.

[61] Li Z., Zhao Z., Zhou Z. (2018). Simultaneous separation and purification of five polymethoxylated flavones from “dahongpao” tangerine (*Citrus tangerina tanaka*) using macroporous adsorptive resins combined with prep-HPLC. Molecules.

[62] Yang Y., Yang S., Xu S., Zhang M., Li C., Li Z., Li Y., Peng L. (2024). Identification of candidate genes involved in scoparone biosynthesis in citrus fruit through transcriptome analysis stimulated by salicylic acid. Postharvest Biol. Technol..

[63] Yamaga I., Nakamura S. (2022). Penicillium growth inhibition, fruit decay reduction, and polymethoxyflavones and scoparone induction in satsuma mandarin irradiated with ultraviolet-A light-emitting diodes. Sci. Hortic..

[64] Lust S., Vanhoecke B., Van Gele M., Philippe J., Bracke M., Offner F. (2010). The flavonoid tangeretin activates the unfolded protein response and synergizes with imatinib in the erythroleukemia cell line K562. Mol. Nutr. Food Res..

[65] Boye A., Ahmad I., Fakhri S., Hussain Y., Khan H. (2021). Incipient citrus polymethoxylated flavone Tangeretin as anticancer drug candidate: Mechanistic insights, limitations and possible solutions. Adv. Cancer Biol. Metastasis.

[66] Yen J.H., Lin C.Y., Chuang C.H., Chin H.K., Wu M.J., Chen P.Y. (2020). Nobiletin Promotes Megakaryocytic Differentiation through the MAPK/ERK-Dependent EGR1 Expression and Exerts Anti-Leukemic Effects in Human Chronic Myeloid Leukemia (CML) K562 Cells. Cells.

[67] Hong M., Zhu H., Liu W., Zhang P., Yu S., Gao Q., Shen G., Li B., Wang G. (2025). Scoparone suppresses proliferation and cell cycle of hepatocellular carcinoma cells via inhibiting AKT/GSK-3β/cyclin D1 signaling pathway. Transl. Cancer Res..

[68] Ling V.Y., Straube J., Godfrey W., Haldar R., Janardhanan Y., Cooper L., Bruedigam C., Cooper E., Tavakoli Shirazi P., Jacquelin S. (2023). Targeting cell cycle and apoptosis to overcome chemotherapy resistance in acute myeloid leukemia. Leukemia.

[69] Paupert J., Mansat-De Mas V., Demur C., Salles B., Muller C. (2008). Cell-surface MMP-9 regulates the invasive capacity of leukemia blast cells with monocytic features. Cell Cycle.

[70] Das S., Amin S.A., Jha T. (2021). Inhibitors of gelatinases (MMP-2 and MMP-9) for the management of hematological malignancies. Eur. J. Med. Chem..

[71] Xian J., Shao H., Chen X., Zhang S., Quan J., Zou Q., Jin H., Zhang L. (2016). Nucleophosmin Mutants Promote Adhesion, Migration and Invasion of Human Leukemia THP-1 Cells through MMPs Up-regulation via Ras/ERK MAPK Signaling. Int. J. Biol. Sci..

[72] Travaglino E., Benatti C., Malcovati L., Della Porta M.G., Galli A., Bonetti E., Rosti V., Cazzola M., Invernizzi R. (2008). Biological and clinical relevance of matrix metalloproteinases 2 and 9 in acute myeloid leukaemias and myelodysplastic syndromes. Eur. J. Haematol..

[73] Asnafi A.A., Bagheri M., Zibara K., Behzad M.M., Shahrabi S. (2019). Expression and activity of matrix Metalloproteinases in leukemia. J. Pediatr. Hematol. Oncol..

[74] Huang X., Qi L., Lu W., Yang G., Chen Y., Zhang R., Rao J., Ji D., Huang R., Chen G. (2017). miRNA-301a induces apoptosis of chronic myelogenous leukemia cells by directly targeting TIMP2/ERK1/2 and AKT pathways. Oncol. Rep..

[75] Liang B., Yin J.J., Zhan X.R. (2015). MiR-301a promotes cell proliferation by directly targeting TIMP2 in multiple myeloma. Int. J. Clin. Exp. Pathol..

[76] Lee Y.Y., Lee E.J., Park J.S., Jang S.E., Kim D.H., Kim H.S. (2016). Anti-Inflammatory and Antioxidant Mechanism of Tangeretin in Activated Microglia. J. Neuroimmune Pharmacol..

[77] Cheng H.L., Hsieh M.J., Yang J.S., Lin C.W., Lue K.H., Lu K.H., Yang S.F. (2016). Nobiletin inhibits human osteosarcoma cells metastasis by blocking ERK and JNK-mediated MMPs expression. Oncotarget.

[78] Sarker S.D., Nahar L. (2012). An introduction to natural products isolation. Nat. Prod. Isol..

[79] Musumeci L., Russo C., Schumacher U., Lombardo G.E., Maugeri A., Navarra M. (2024). The pro-differentiating capability of a flavonoid-rich extract of *Citrus bergamia* juice prompts autophagic death in THP-1 cells. Sci. Rep..

[80] Ferlazzo N., Cirmi S., Russo M., Trapasso E., Ursino M.R., Lombardo G.E., Gangemi S., Calapai G., Navarra M. (2016). NF-kappaB mediates the antiproliferative and proapoptotic effects of bergamot juice in HepG2 cells. Life Sci..

[81] Russo C., Maugeri A., De Luca L., Gitto R., Lombardo G.E., Musumeci L., De Sarro G., Cirmi S., Navarra M. (2022). The SIRT2 Pathway Is Involved in the Antiproliferative Effect of Flavanones in Human Leukemia Monocytic THP-1 Cells. Biomedicines.

[82] Russo C., Maugeri A., Albergamo A., Dugo G., Navarra M., Cirmi S. (2023). Protective Effects of a Red Grape Juice Extract against Bisphenol A-Induced Toxicity in Human Umbilical Vein Endothelial Cells. Toxics.

[83] Zhang W., Wang J., Wang Y., Dong F., Zhu M., Wan W., Li H., Wu F., Yan X., Ke X. (2015). B7-H3 silencing by RNAi inhibits tumor progression and enhances chemosensitivity in U937 cells. OncoTargets Ther..

