# Supplementary material for: Mechanisms of Antiproliferative Effects of Nobiletin, Scoparone, and Tangeretin Isolated from Citrus reticulata Peel Dichloromethane Extract in Acute Myeloid Leukemia Cells
Source: Int J Mol Sci. 2026 Jan 27;27(3):1256. doi: 10.3390/ijms27031256 (PMC12898205; doi:10.3390/ijms27031256)
Supplement: Supplementary file 1 [file ijms-27-01256-s001.zip › ijms-4066000-supplementary.pdf]

International Journal of Molecular Sciences

Paper

Supplementary materials

1. Characterization of A compound as the flavonoid nobiletin

Compound A was isolated from F_{DCM} by PTLC and its ^{1}HNMR spectrum (600 MHz, $CDCl_3$) was shown in Figure S1. This included signals typical of a polymethoxyflavone skeleton, as revealed by protons of four methoxy groups linked to ring A: δ ppm 3.95 (3H, *s*, 5-OCH3), 3.95 (3H, *s*, 6-OCH3), 4.02 (3H, *s*, 8-OCH3) and 4.1 (3H, *s*, 7-OCH3). In addition, the presence of other two methoxy groups were associated with ring B at δ ppm 3.97 (3H, *s*, 3′-OCH3) and 3.98 (3H, *s*, 4′-OCH3). In ring B *ortho*-coupled protons were related to signals at δ ppm 7.41 (1H, *d*, *J*=2, H-2′) and 7.02 (1H, *d*, *J*=9, H-5′), whereas *ortho-meta* coupled aromatic protons produced the signal at δ ppm 7.57 (1H, *dd*, J=2, 8.4, H-6′). Finally, a sharp singlet indicated the olefinic proton in the ring C at δ ppm 6.62 (1H, *s*, H-3). Other signals obtained on ^{1}HNMR spectrum were not considered since they represented impurities.

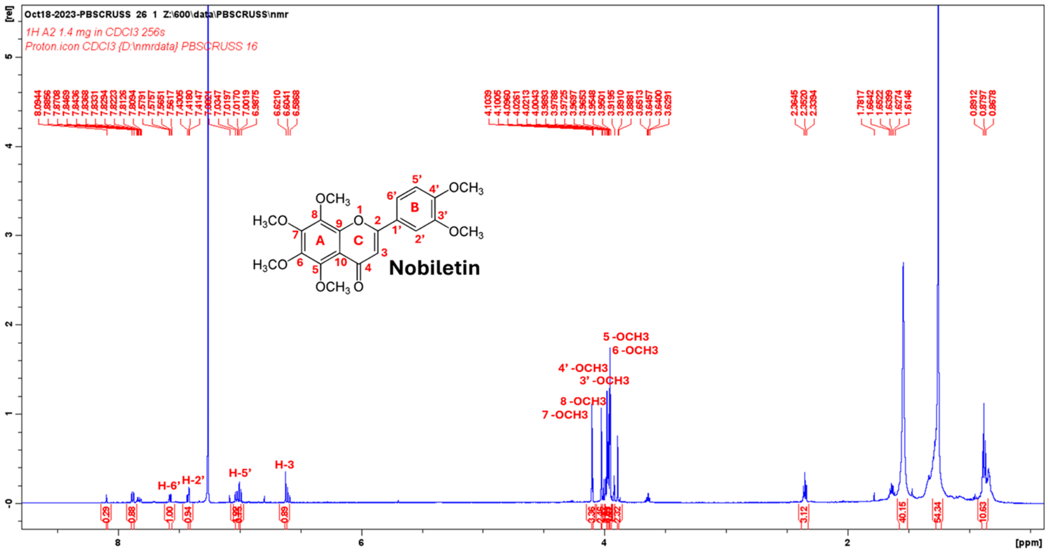

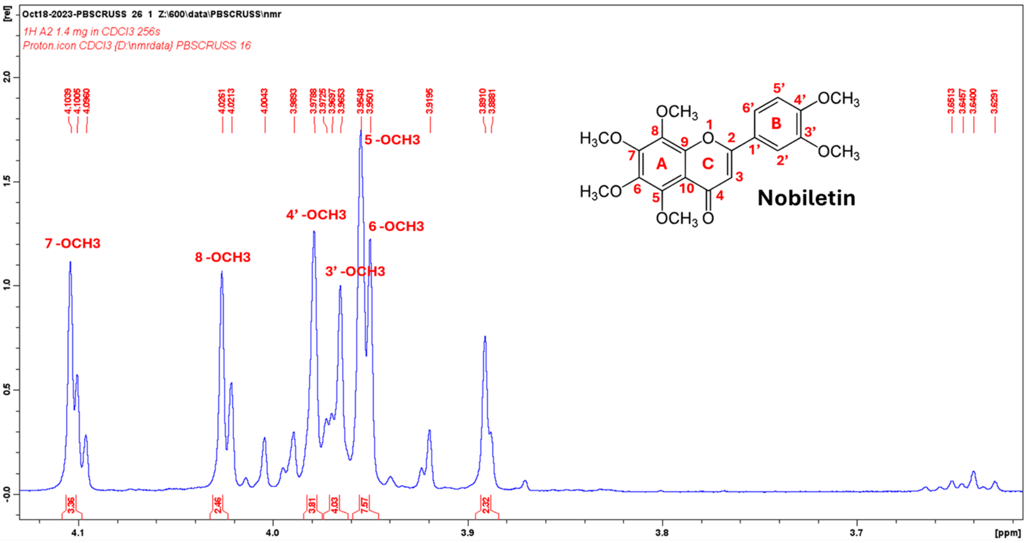

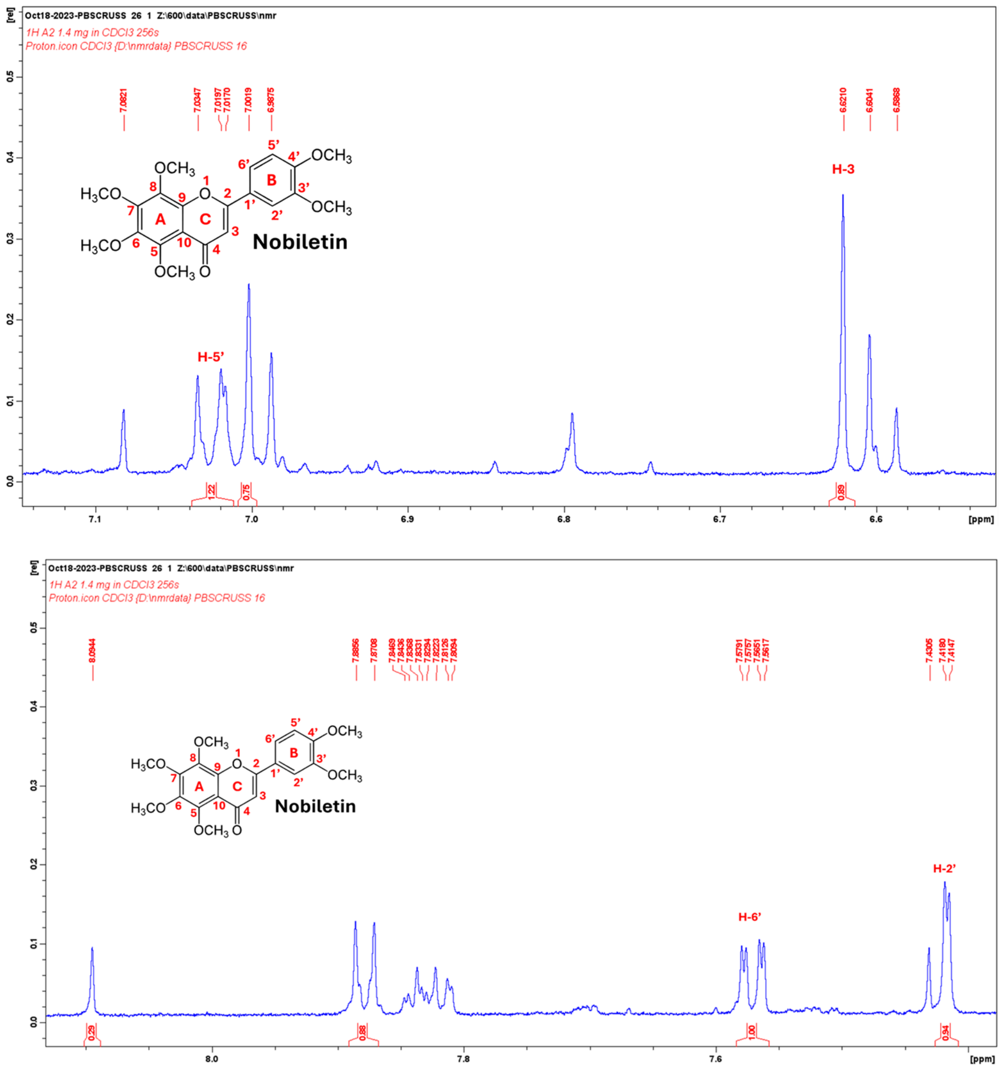

Figure S1. ¹H NMR spectrum of compound A and related expansions. Compound A was identified as nobiletin according to its ¹H NMR spectrum (600 MHz, $CDCl_3$). The whole ¹H NMR spectrum and related expansions are shown.

2. Characterization of B compound as the coumarin scoparone

Compound B was isolated from F_{DCM} by PTLC. Its ¹HNMR spectrum (600 MHz, $CDCl_3$) was reported in Figure S2. Compound B was not totally pure; the proton spectrum between 3.9 ppm to 8.1 ppm is the range useful for its identification (Figure S2). Signals typical of coumarin were detected at δ ppm 7.63 (1H, *d*, *J*=9.5, H-1), 6.30 (1H, *d*, *J*=9.5, H-2) and two aromatic protons at δ ppm 6.85 (1H, *s*, H-5) and 6.85 (1H, *s*, H-8). Other two proton signals were ascribed to the presence of methoxy groups on aromatic ring at δ ppm 3.92 (3H, *s*, 6-OCH_3) and 3.95 (3H, *s*, 7-OCH_3). Other signals detected by proton analysis were not considered relevant for identification.

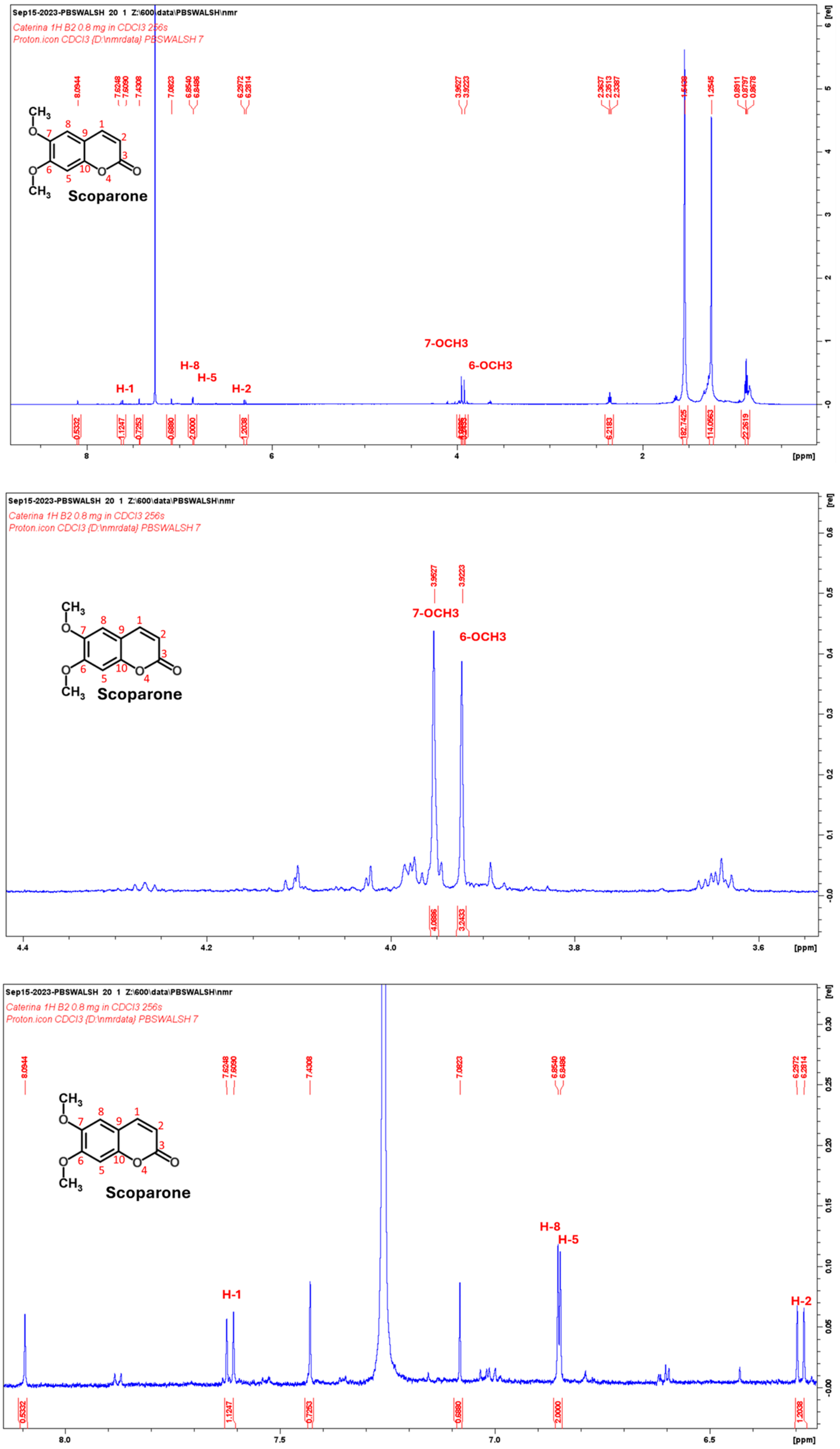

Figure S2. ^{1}H NMR spectrum of compound B and related expansions. Compound B was identified as scoparone according to its ^{1}H NMR spectrum (600 MHz, $CDCl_3$). The whole proton spectrum as well as related NMR expansions were reported.

3. Characterization of D compound as the flavonoid tangeretin

Compound D was isolated from Fdcm by PTLC. Its ^{1}HNMR spectrum (600 MHz, $CDCl_3$) was shown in Figure S3. The polymethoxyflavone skeleton was detected by proton signals of methoxy groups bound to ring A at δ ppm 3.95 (6H, *s*, 5-OCH3 and 6-OCH3), 4.10 (3H, *s*, 7-OCH3) and 4.02 (3H, *s*, 8-OCH3). In same ppm region another

proton signal was assigned to methoxy group linked to B ring at δ ppm 3.9 (3H, *s*, 4′-OCH3). In same ring *ortho* protons signals were assigned at δ ppm 7.89 (2H, *m*, H-2′ and H-6′) whereas *meta* protons corresponded to signals at δ ppm 7.03 (2H, *m*, H-3′ and H-5′). A sharp singlet indicated the olefinic proton in the ring C at δ ppm 6.60 (1H, *s*, H-3). Other signals were not considered since they represented solvent impurities.

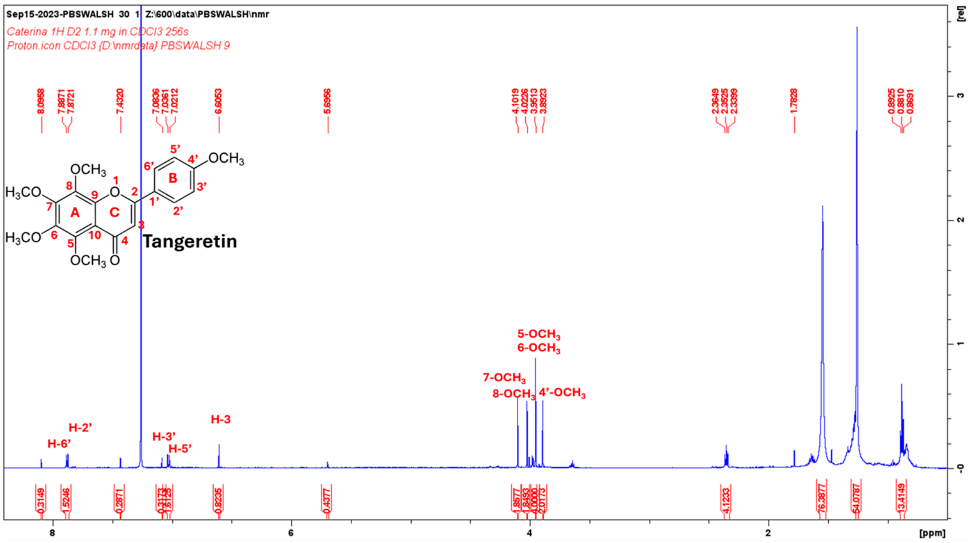

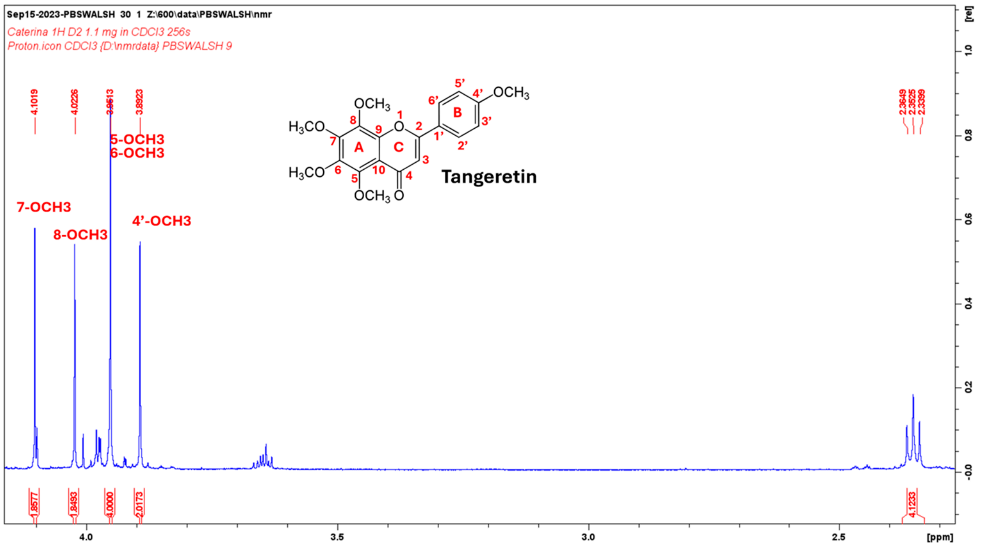

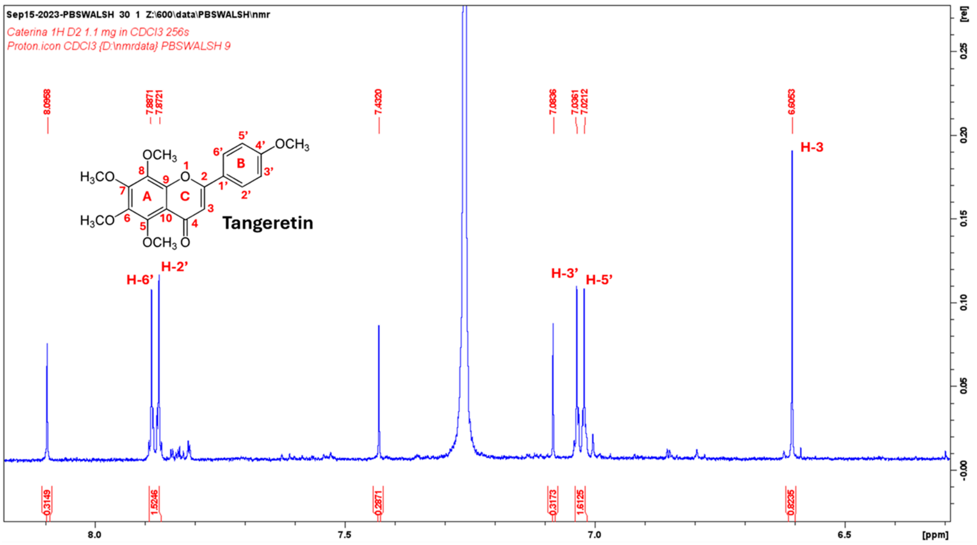

Figure S3. ^{1}H NMR spectrum of compound D and related expansions. Compound D was identified as tangeretin according to its ^{1}H NMR spectrum (600 MHz, CDCl3). The whole proton spectrum as well as NMR expansions are reported.
